# *Arabidopsis thaliana DM2h (R8)* within the Landsberg *RPP1-like Resistance* Locus Underlies Three Different Cases of *EDS1*-Conditioned Autoimmunity

**DOI:** 10.1371/journal.pgen.1005990

**Published:** 2016-04-15

**Authors:** Johannes Stuttmann, Nora Peine, Ana V. Garcia, Christine Wagner, Sayan R. Choudhury, Yiming Wang, Geo Velikkakam James, Thomas Griebel, Ruben Alcázar, Kenichi Tsuda, Korbinian Schneeberger, Jane E. Parker

**Affiliations:** 1 Department of Plant-Microbe Interactions, Max-Planck Institute for Plant Breeding Research, Cologne, Germany; 2 Department of Genetics, Martin Luther University Halle (Saale), Halle, Germany; 3 Department of Plant Developmental Biology, Max-Planck Institute for Plant Breeding Research, Cologne, Germany; 4 Department of Natural Products, Plant Biology and Soil Science, Faculty of Pharmacy, University of Barcelona, Barcelona, Spain; The Sainsbury Laboratory, UNITED KINGDOM

## Abstract

Plants have a large panel of nucleotide-binding/leucine rich repeat (NLR) immune receptors which monitor host interference by diverse pathogen molecules (effectors) and trigger disease resistance pathways. NLR receptor systems are necessarily under tight control to mitigate the trade-off between induced defenses and growth. Hence, mis-regulated NLRs often cause autoimmunity associated with stunting and, in severe cases, necrosis. Nucleocytoplasmic ENHANCED DISEASE SUSCEPTIBILITY1 (EDS1) is indispensable for effector-triggered and autoimmune responses governed by a family of Toll-Interleukin1-Receptor-related NLR receptors (TNLs). EDS1 operates coincidently or immediately downstream of TNL activation to transcriptionally reprogram cells for defense. We show here that low levels of nuclear-enforced EDS1 are sufficient for pathogen resistance in *Arabidopsis thaliana*, without causing negative effects. Plants expressing higher nuclear EDS1 amounts have the genetic, phenotypic and transcriptional hallmarks of TNL autoimmunity. In a screen for genetic suppressors of nuclear EDS1 autoimmunity, we map multiple, independent mutations to one gene, *DM2h*, lying within the polymorphic *DANGEROUS MIX2* cluster of TNL *RPP1-like* genes from *A*. *thaliana* accession Landsberg *erecta* (L*er*). The *DM2* locus is a known hotspot for deleterious epistatic interactions leading to immune-related incompatibilities between *A*. *thaliana* natural accessions. We find that *DM2h*^*Ler*^ underlies two further genetic incompatibilities involving the *RPP1-like*^Ler^ locus and *EDS1*. We conclude that the DM2h^Ler^ TNL protein and nuclear EDS1 cooperate, directly or indirectly, to drive cells into an immune response at the expense of growth. A further conclusion is that regulating the available EDS1 nuclear pool is fundamental for maintaining homeostatic control of TNL immune pathways.

## Author Summary

Plants tune their cellular and developmental programs to different environmental stimuli. Central players in the plant biotic stress response network are intracellular NLR receptors which intercept specific disease-inducing molecules (effectors) produced by pathogenic microbes. Variation in NLR gene repertoires between plant genetic lines is driven by pathogen selection pressure. One evolutionary question is how new, functional NLRs are assembled within a plant genome without mis-activating defense pathways, which can have strong negative effects on growth and fitness. This study focuses on a large, polymorphic sub-class of NLR receptors called TNLs present in dicotyledenous plant lineages. TNL receptors confer immunity to a broad range of pathogens. They also frequently underlie autoimmunity caused by their mis-regulation or deleterious allelic interactions with other genes in crosses between different genetic lines (hybrid incompatibility, HI). TNL pathogen-triggered and autoimmune responses require the conserved nucleocytoplasmic protein EDS1 to transcriptionally reprogram cells for defense. We discover in *Arabidopsis thaliana* that high levels of nuclear-enriched EDS1 induce transcriptional activation of defenses and growth inhibition without a pathogen effector stimulus. In a mutational screen, we identify one rapidly evolving TNL gene, *DM2h*^Ler^, as a driver of nuclear EDS1 autoimmunity. *DM2h*^Ler^ also contributes to two separate cases of *EDS1*-dependent autoimmunity. Genetic cooperation between *DM2h*^Ler^ and *EDS1* suggests a functional relationship in the transcriptional feed-forward regulation of defense pathways.

## Introduction

In plants, receptors that sense pathogen attack are central players in the biotic stress signaling network. Receptor activation triggers innate immunity pathways to protect cells and tissues from disease. In a first line of defense, surface pattern recognition receptors (PRRs) bind microbial molecules to activate disease resistance programs leading to pattern-triggered immunity (PTI). A second critical immunity layer is mediated by intracellular nucleotide-binding/leucine-rich-repeat (NLR) receptors that recognize virulence factors (called effectors) which are delivered by pathogen strains to dampen PTI and promote disease [1]. Structural counterparts of plant NLRs called NOD-LRR (nucleotide-binding/oligomerization-domain/leucine-rich-repeat) receptors also sense pathogen interference in mammalian systems [2, 3]. NLR and NOD-LRR proteins are ATP-driven molecular switches which become stimulated by direct binding of an effector molecule or effector modifications of an NLR-monitored host target [4, 5]. In plants, NLR activation induces a robust resistance response called effector-triggered immunity (ETI) involving the amplification of PTI-related transcriptional programs and, often, host cell death at infection sites (a hypersensitive response, HR) [6].

NLRs are among the most rapidly evolving plant genes [7–9], and expansion in NLR gene number and diversity, as paralogs within complex loci or allelic variants in different genotypes, is in part driven by pathogen effector pressure [10–13]. Receptor monitoring (or guarding) of important defense hubs that are targeted by multiple pathogen effectors probably further increases NLR recognition space [14–17]. Nevertheless, the rapid evolution of NLR genes creates potentially dangerous molecules if activated in the absence of a pathogen effector stimulus [4, 18].

Loss of NLR homeostasis caused by mutation, mis-expression or disturbance of NLR-monitored co-factors leads to autoimmunity. Plant autoimmune backgrounds display constitutive defense gene expression and varying degrees of stunting, necrosis and reduced reproductive fitness [19]. As in ETI, NLR autoimmune phenotypes are often conditional on temperature with high temperatures (25–28°C) suppressing disease resistance, transcriptional activation of defense pathways and HR-related cell death [19–21]. Temperature-conditioned autoimmunity can also arise in the progeny of inter- or intra-specific crosses between different genetic backgrounds to produce immune-related hybrid incompatibility (HI) (known also as hybrid necrosis) [19, 22]. HI is caused by deleterious epistatic interactions between two or more loci that have diverged through genetic drift or selection in the different parental lineages [23–25]. Mapping of the causal interacting genes or allelic forms in several cases of temperature-conditioned HI shows that many are in NLR or immune-related loci [18, 22, 25–29]. Therefore, HI might expose altered NLR regulation and/or associations with monitored co-factors as immunity systems evolve.

Effector-activated NLR receptors connect to a conserved basal resistance network to mobilize ETI defense pathways [6]. Although the downstream events are not well understood, signals in ETI ultimately converge on the nuclear transcription machinery to boost PTI-related defense programs [6]. A major NLR subclass in dicotyledenous species has an N-terminal Toll-Interleukin1-receptor (TIR) domain (referred to as TNLs or TIR-NB-LRRs) [9, 30] and requires the nucleocytoplasmic, lipase-like protein ENHANCED DISEASE SUSCEPTIBILITY1 (EDS1) for all measured ETI and autoimmunity outputs [21, 31–34]. Interactions between EDS1 and TNL proteins suggested that EDS1 provides an immediate link between TNLs and downstream resistance pathways [35–37]. Importantly, EDS1 nuclear accumulation was found to be necessary for *A*. *thaliana* basal immunity against virulent pathogen strains and TNL-triggered ETI, consistent with a central EDS1 role in transcriptional reprogramming of cells for defense [21, 32, 38]. Analysis of *A*. *thaliana* transgenic plants in which EDS1 was mis-localized to the cytoplasm or its nucleocytoplasmic trafficking disturbed, suggested also that the EDS1 cytoplasmic pool contributes to resistance [38, 39].

Unlike many mis-regulated NLRs, over-accumulation of functional, nucleocytoplasmic *A*.*thaliana* EDS1 does not cause autoimmunity [38, 40]. Here, we investigated the consequences of restricting *A*. *thaliana* EDS1 to the nuclear compartment. Our analysis shows that a low-level EDS1 nuclear pool, operating with signaling partners, is sufficient for mediating *A*. *thaliana* basal and TNL immunity without deleterious consequences for the plant. However, raising nuclear EDS1 amounts above a certain threshold leads to autoimmunity with many features of a deregulated TNL immune response. In a screen for genetic suppressors, we discover that the nuclear EDS1 autoimmune phenotype depends on presence of the ‘*DANGEROUS MIX2*’ (*DM2*) *RPP1-like* TNL gene cluster. The *DM2* locus is a hotspot for genes underlying immune-related HI. In our case, a cluster of eight *RPP1-like* TNL genes linked to an *eds1* deletion mutation had been co-introgressed from *A*. *thaliana* accession Landsberg *erecta* into accession Columbia (Col). We identify one gene, *DM2h*, within the *DM2 RPP1-like*^Ler^ locus as necessary for nuclear EDS1 autoimmunity. We propose that a weak *DM2h*^Ler^ autoactivity which is normally constrained is exposed by nuclear EDS1, producing *EDS1*-dependent defense expression and autoimmunity. A corollary of this damaging co-action between a TNL and nuclear EDS1 is that in wild-type plants, regulating the nuclear EDS1 pool likely helps to maintain TNL immune pathway homeostasis and growth.

## Results

### High levels of nuclear-targeted EDS1 lead to autoimmunity

**Fig 1:**
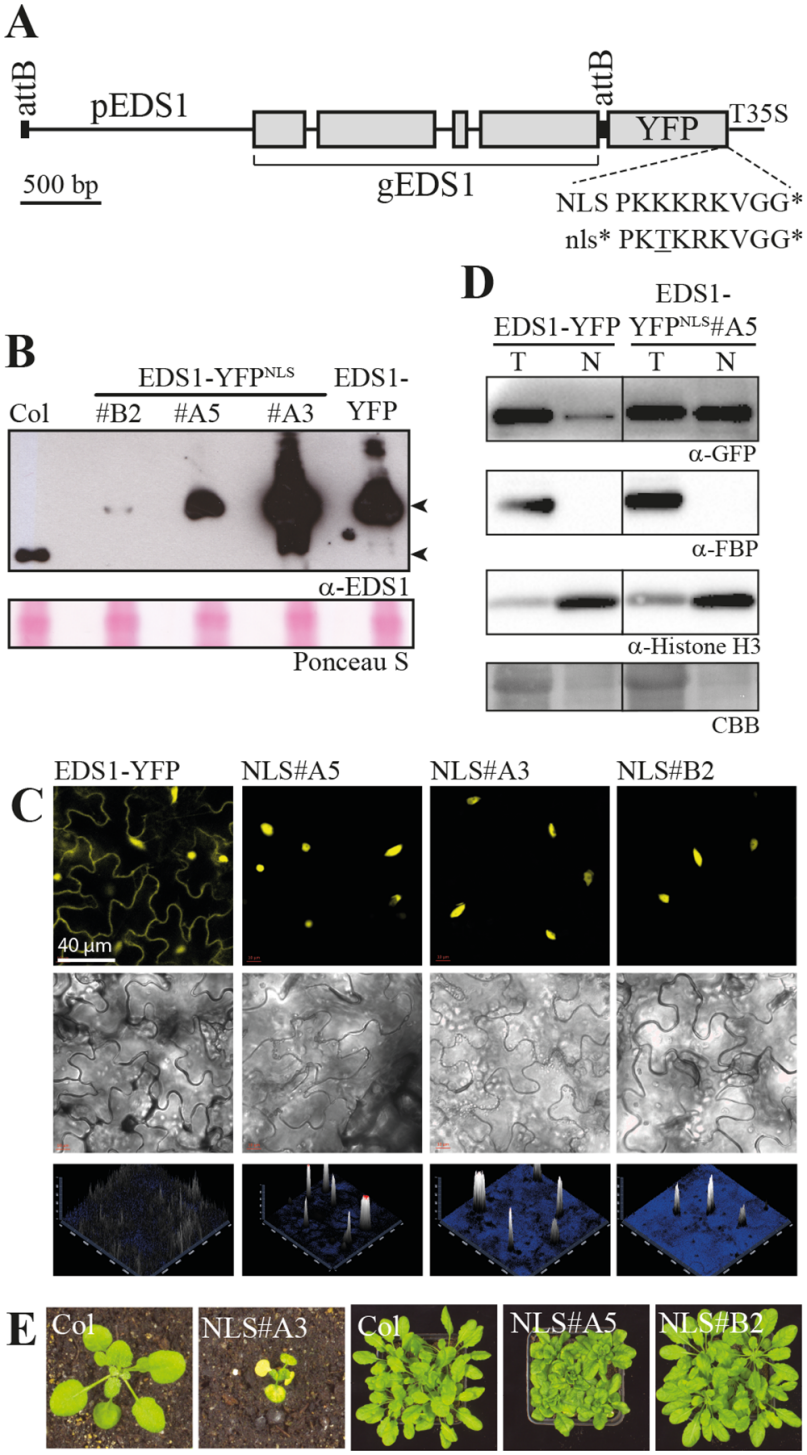
Nuclear-enforced expression of EDS1 in transgenic *A*. *thaliana*. A. Schematic drawing of the EDS1-YFP^NLS/nls^* expression cassette used for *A*. *thaliana* Col *eds1-2* transformation. gEDS1, EDS1 genomic sequence with exons shown as boxes and introns as lines; pEDS1, EDS1 promoter; T35S, CaMV35S terminator; attB, recombination sites; PKK/TKRKRVG*, SV40 NLS and non-functional nls* variant with stop fused to the last codon of YFP. B. Immunoblot analysis of total protein extracts from 3-week-old plants separated by SDS-PAGE and probed with α-EDS1 antibody. Positions of endogenous EDS1 and transgenic EDS1-YFP are marked. Transgenic lines were generated in the Col *eds1-2* background. Ponceau S staining of the membrane is shown as a loading control. C. Confocal live cell imaging of representative leaf epidermal cells from EDS1-YFP and EDS1-YFP^NLS^ transgenic plants. YFP channel, bright field image and an intensity projection of the YFP channel are shown. D. Nuclear accumulation of EDS1-YFP and EDS1-YFP^NLS^ analyzed by biochemical fractionation. 4-week-old plants before the onset of developmental defects were used for fractionation. Equal amounts of total protein extracts (T), and nuclear fractions (N) were separated by SDS-PAGE and used for immunodetection. A Coomassie-stained section of the membrane is shown as loading control (CBB). Black line indicates splicing of additional lanes from the same membrane. E. Development of macroscopic growth phenotypes in transgenic EDS1-YFP^NLS^ lines #A3, #A5 and #B2 compared to wild-type Col after 3 and 6 weeks cultivation at 22°C, respectively.

We tested whether increased targeting of EDS1 to nuclei affects its disease resistance activity. For this, *A*. *thaliana* stable transgenic lines expressing genomic *EDS1* under control of its native promoter and fused to a C-terminal yellow fluorescent protein (YFP) tag and SV40 nuclear localization signal (NLS) were generated in an *eds1-2* deletion mutant in accession Col-0 (Col) (Fig 1A). The *eds1-2* mutation had been introgressed originally from accession Landsberg *erecta* (L*er*) over eight backcrosses because Col contains a tandem duplication of two functional *EDS1* genes [41]. Three independent EDS1-YFP^NLS^ lines (#A3, #A5 and #B2) were taken to homozygosity and tested alongside a previously characterized Col *eds1-2* transgenic line expressing functional, genomic *EDS1-YFP* [38]. EDS1-YFP protein accumulation in leaf extracts of the different transgenic lines was compared to that of native EDS1 in Col by immunoblotting with anti-EDS1 antibodies. The EDS1-YFP^NLS^ protein levels ranged from lower than wild-type EDS1 (in EDS1-YFP^NLS^ line #B2) to higher than wild-type EDS1 (EDS1-YFP^NLS^ line #A5), with highest accumulation in EDS1-YFP^NLS^ line #A3 (Fig 1B). Accumulation of EDS1-YFP (without an additional NLS) was intermediate between that of EDS1-YFP^NLS^ lines #A5 and #A3 (Fig 1B). Confocal laser scanning microscopy of leaf epidermal cells showed that EDS1-YFP distributed in the cytoplasm and nucleus, as expected [38], whereas EDS1-YFP^NLS^ was detected only in nuclei in lines #B2, #A5 and #A3 (Fig 1C). Biochemical purification of nuclei from leaf tissues showed that there was strong nuclear enrichment of EDS1 protein in the EDS1-YFP^NLS^ line #A5 compared to EDS1-YFP (Fig 1D).

Growth of EDS1-YFP and EDS1-YFP^NLS^ #B2, #A5 and #A3 plants in soil under short-day conditions (10 h light period at 22°C) was monitored over several weeks. EDS1-YFP and the EDS1-YFP^NLS^ low expressor line #B2 were undistinguishable from wild-type Col or Col *eds1-2* (Figs 1E and S1A). By contrast, EDS1-YFP^NLS^ #A3 seedlings became stunted and chlorotic after the first true leaves emerged at ~ 2 weeks and were dead at 4 weeks (Figs 1E and S1A). EDS1-YFP^NLS^ #A5 plants displayed stunting, curling of leaves and chlorosis from 4–5 weeks but remained viable and partially fertile (Figs 1E and S1A). The developmental defects of EDS1-YFP^NLS^ lines #A3 and #A5 co-segregated with the T-DNA selection marker. Also, the T-DNA insertion in EDS1-YFP^NLS^ #A3 mapped to the first exon of At4g28490, in which an insertion mutation (in the *haesa (hae)* single mutant) does not have a visible phenotype [42]. These results suggest that increased EDS1 nuclear localization or an imbalance in EDS1 nucleocytoplasmic partitioning, rather than EDS1 over expression, leads to EDS1 dose-dependent growth defects. We also generated Col *eds1-2* transgenic lines expressing EDS1-YFP fused to a mutated, inactive NLS (Figs 1A and S1) [43]. No line was found that expressed EDS1-YFP^nls^ protein as highly as EDS1-YFP^NLS^ in line #A5. Two EDS1-YFP^nls^ lines (nls*#α5 and nls*#β5) were selected that had moderately high EDS1-YFP expression (S1B Fig). These showed a nucleocytoplasmic distribution of EDS1-YFP (S1C Fig) and grew normally at 22°C (S1D Fig).

**Fig 2:**
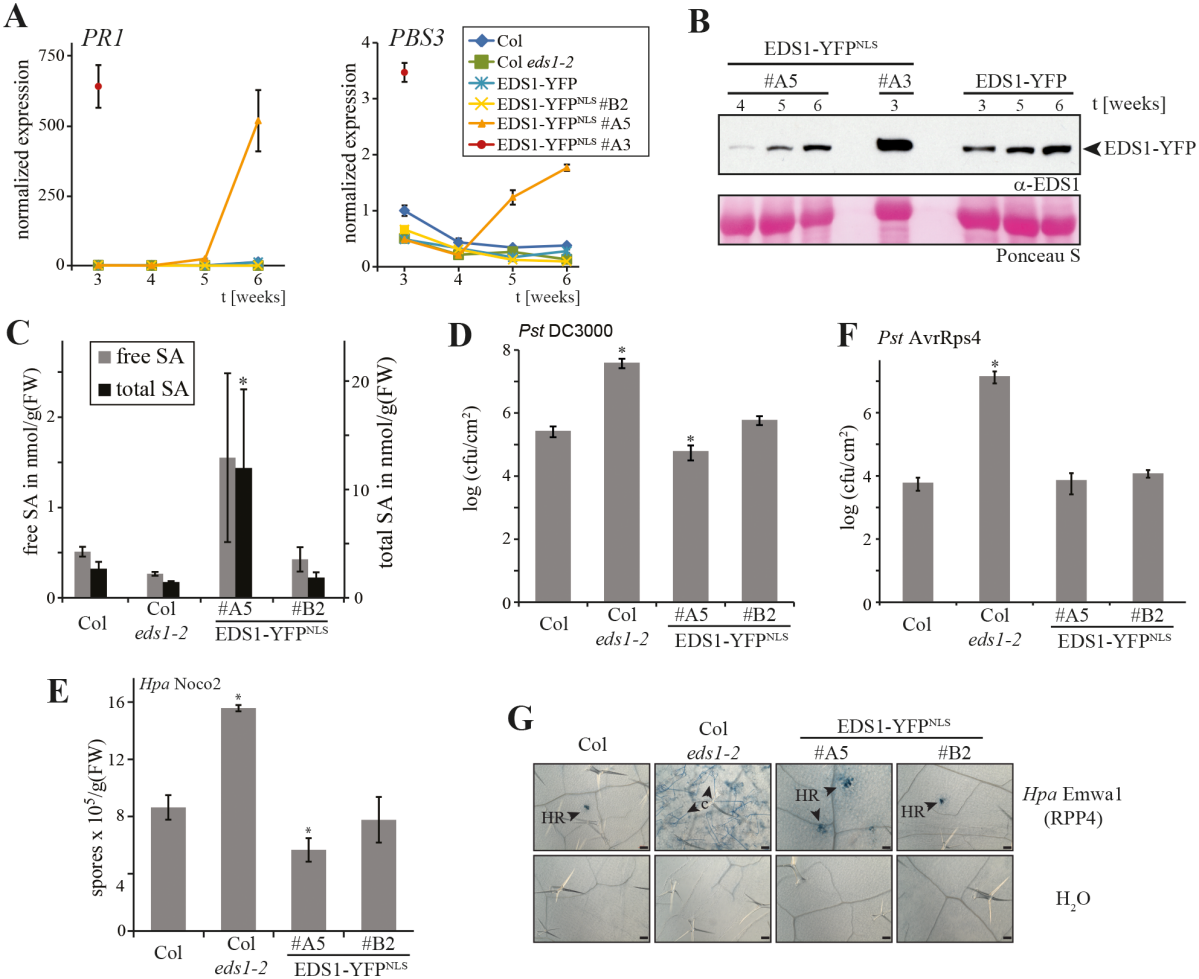
Disease resistance responses in EDS1-YFP^NLS^ transgenic plants. A. *PR1* and *PBS3* marker gene expression normalized to *UBQ10* in EDS1-YFP and EDS1-YFP^NLS^ transgenic lines or control plants monitored by qRT-PCR over 3–6 weeks. Error bars represent standard deviation of three technical replicates. B. Immunoblot analysis of total protein extracts from the plants used in (A) separated by SDS-PAGE and probed with α-EDS1 antibody. Ponceau S staining of the membrane is shown as a loading control. C. Accumulation of free and total salicylic acid (SA) in 5-week-old EDS1-YFP^NLS^ transgenic lines #A5 and #B2 and control plants. SA was measured by GC-MS using three replicates with standard deviations shown. *, significant difference to Col in a Student’s t-test, p < 0.05. D. Bacterial growth of *Pst* DC3000 on EDS1-YFP^NLS^ transgenic lines #A5 and #B2 and control plants at 3 dpi. 5-week-old plants were spray-inoculated and bacterial titers in leaves determined. Error bars indicate standard error of ≥ 10 biological replicates. *, significant difference to Col in a Student’s t-test, p < 0.05. E. Numbers of conidiospore at 7 dpi on leaves of 3-week-old plants inoculated with virulent *Hpa* isolate Noco2. Standard deviation of four biological replicates is shown. *, significant difference to Col in a Student’s t-test, p < 0.05. F. Bacterial growth of *Pst* AvrRps4 on EDS1-YFP^NLS^ transgenic lines #A5 and #B2 and control plants at 3 dpi, as performed in (D). Error bars indicate standard error of five biological replicates *, significant difference to Col in a Student’s t-test, p < 0.05. G. Representative micrographs of Trypan blue-stained first true leaves of 3-week-old EDS1-YFP^NLS^ transgenic lines #A5 and #B2 and control plants 5 d after spray-inoculation with avirulent *Hpa* isolate Emwa1. Water-sprayed plants were examined alongside for evidence of spontaneous necrotic lesion formation. HR, hypersensitive response; c—conidiophores. Scale bar = 100 μm.

Because the developmental phenotypes in EDS1-YFP^NLS^ lines #A3 and #A5 resemble *A*. *thaliana* autoimmunity backgrounds we measured expression of the *EDS1*-dependent defense marker genes *PATHOGENESIS RELATED1* (*PR1*) and *AvrPphB SUSCEPTIBLE3* (*PBS3*) in EDS1-YFP^NLS^ transgenic and control lines. *PR1* and *PBS3* expression remained low in Col, Col *eds1-2* and the phenotypically normal EDS1-YFP or EDS1-YFP^NLS^ #B2 lines over a 3–6 week growth period (Fig 2A). From ~ 4 weeks on, *PR1* and *PBS3* expression increased in EDS1-YFP^NLS^ line #A5 (Figs 2A and S1E), consistent with the appearance of macroscopic growth defects. High *PR1* and *PBS3* expression was also detected in 3-week-old dying EDS1-YFP^NLS^ #A3 plants (Fig 2A). By contrast, the EDS1-YFP-nls lines *#α5 and *#β5 did not have elevated *PR1* expression (S1F Fig).

A gradual increase in EDS1 total protein accumulation over 3–6 weeks development was detected in both the EDS1-YFP and EDS1-YFP^NLS^ lines (Fig 2B), suggesting that there is a general rise in EDS1 steady state levels as plants age, regardless of EDS1 nucleocytoplasmic or nuclear distribution. Total and free SA levels were unchanged in 5-week-old EDS1-YFP^NLS^ #B2, Col and Col *eds1-2* plants, but were high in line EDS1-YFP^NLS^ #A5 (Fig 2C). Hence, during development, accumulation of nuclear EDS1 in EDS1-YFP^NLS^ lines #A3 and #A5 appears to reach a threshold for causing defense gene activation and disturbed growth. These results show that nuclear EDS1-YFP^NLS^ in line #A5, and more acutely in #A3, has the capacity to transcriptionally activate defense pathways in the absence of a pathogen stimulus.

### A low level of nuclear-enriched EDS1 confers basal and effector-triggered immunity

We tested whether EDS1 targeted to nuclei is sufficient to confer basal disease resistance by spray-infecting leaves with the virulent bacterial pathogen *Pseudomonas syringae* pv. *tomato* strain DC3000 (*Pst* DC3000). As expected, *Pst* DC3000 growth was higher at 3 d post-infection (3 dpi) in Col *eds1-2* than in wild-type Col leaves, indicative of a loss of basal resistance in Col *eds1-2* (Fig 2D). The *eds1-2* defect was fully complemented in EDS1-YFP^NLS^ #B2 expressing low levels of EDS1-YFP^NLS^ (Figs 1B and 2D). *Pst* DC3000 growth was marginally reduced on EDS1-YFP^NLS^ #A5 compared to wild-type Col plants (Fig 2D). Similar resistance trends were observed in these transgenic lines in response to infection by a virulent oomycete pathogen, *Hyaloperonospora arabidopsidis* (*Hpa*, isolate Noco2) (Fig 2E). We then tested whether nuclear-enriched EDS1 functions in ETI by inoculating plants with *Pst* DC3000 delivering the Type-III secreted effector AvrRps4 (*Pst* AvrRps4), or with an incompatible *Hpa* isolate, Emwa1. In accession Col, AvrRps4 is recognized by the nuclear TNL receptor pair RRS1/RPS4 [32, 44–46] and *Hpa* Emwa1 by the TNL receptor RPP4 [47], in *EDS1*-dependent ETI. Accordingly, *Pst* AvrRps4 growth at 3 dpi was restricted in wild-type Col in an *EDS1*-dependent manner (Fig 2F). RRS1/RPS4 ETI against *Pst* AvrRps4 was also fully restored in EDS1-YFP^NLS^ lines #A5 and #B2 (Fig 2F), as well as in EDS1-YFP^nls^ lines *#α5 and *#β5 (S1G Fig). EDS1-YFP^NLS^ #A5 and #B2 restricted *Hpa* Emwa1 growth as efficiently as wild-type Col, with all lines exhibiting a host hypersensitive response (HR) at attempted *Hpa* infection sites, as measured by Trypan Blue (TB)-staining of infected leaves (Fig 2G). As expected, Col *eds1-2* plants were fully susceptible to *Hpa* Emwa1 infection (Fig 2G). No HR lesioning was observed in mock-inoculated EDS1-YFP^NLS^ lines #A5 or #B2, indicating that the host HR is pathogen-triggered (Fig 2G). We concluded that even low levels of nuclear-targeted EDS1, as in EDS1-YFP^NLS^ #B2, are sufficient for *Arabidopsis* basal and TNL-conditioned immunity.

### High temperature suppresses EDS1-YFP^NLS^ autoimmunity but not EDS1 nuclear accumulation

**Fig 3:**
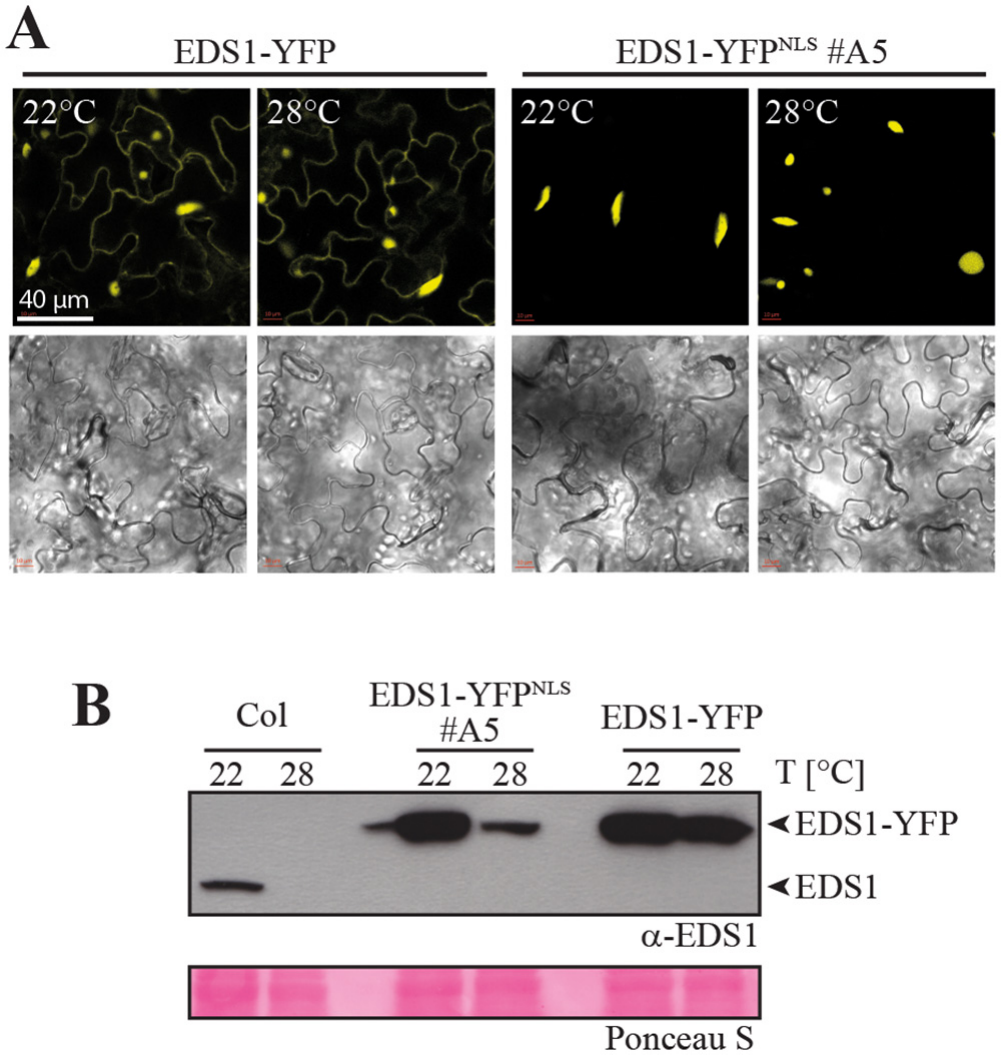
Temperature modulation of EDS1 protein accumulation and localization. A. Confocal live cell images of representative leaf epidermal cells from EDS1-YFP and EDS1-YFP^NLS^ #A5 plants grown at 22°C or 28°C. B. Immunoblot analysis of total protein extracts from 4-week-old plants grown at 22°C or 28°C separated by SDS-PAGE and probed with α-EDS1 antibody. Ponceau S staining of the membrane is shown as loading control.

Because many *Arabidopsis* effector-triggered TNL and autoimmunity phenotypes are attenuated at elevated temperatures, we tested whether high temperature alters EDS1-YFP nuclear accumulation. At 28°C, accumulation of the nucleocytoplasmic TNL proteins tobacco N, *Arabidopsis* RPS4 and SNC1 (SUPPRESSOR OF npr1-1 CONSTITUTIVE1) inside nuclei and *EDS1*-dependent transcriptional reprogramming are reduced [21, 48, 49]. Macroscopic growth defects and enhanced *PR1* expression in EDS1-YFP^NLS^ lines #A3 and #A5 at 22°C were also suppressed when plants were propagated at 28°C (S1A and S1E Fig). Confocal laser scanning microscopy of leaves taken directly from plants grown at 22°C or 28°C showed that the distribution of nucleocytoplasmic EDS1-YFP or nuclear EDS1-YFP^NLS^ fluorescence signals did not change substantially between the two temperature regimes (Fig 3A). Therefore, high temperature suppression of EDS1-YFP^NLS^ autoimmunity in line #A5 is not due to a failure in EDS1 nuclear import. However, steady state levels of EDS1-YFP^NLS^ were lower in plants grown at 28°C compared to 22°C, as monitored on immunoblots with anti-EDS1 antibodies (Fig 3B). A decrease in native EDS1 protein accumulation was also detected in wild-type Col grown at 28°C (Fig 3B). Therefore, growth at 28°C leads to reduced EDS1 protein accumulation regardless of whether EDS1 is confined to the nucleus or free to shuttle between the nucleus and cytoplasm [38]. This is in line with a reported lowering of *EDS1* transcript levels under high temperature conditions [50]. We concluded that suppression of autoimmunity in EDS1-YFP^NLS^ #A5, and probably also #A3 at 28°C (S1A Fig), is caused by reduction of nuclear EDS1 to below a threshold needed to elicit autoimmunity.

### Nuclear *EDS1*-induced autoimmunity requires *PAD4*

**Fig 4:**
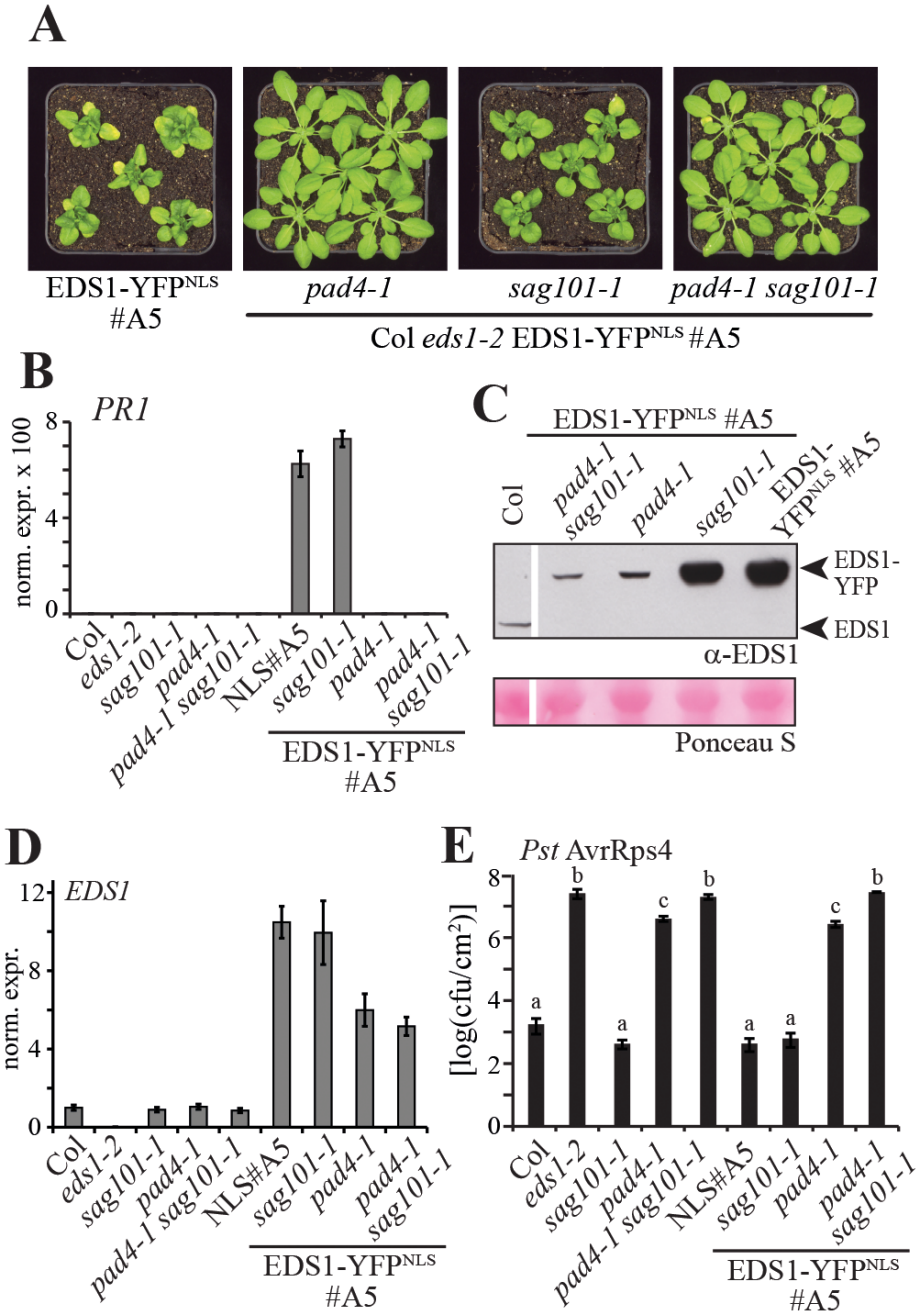
Genetic analysis of nuclear EDS1 autoimmunity. A. Macroscopic growth phenotypes at 22°C of 5-week-old Col *eds1-2* plants containing the EDS1-YFP^NLS^ #A5 transgene and *pad4-1* and/or *sag101-1* mutations, as indicated. B. *PR1* marker gene expression in different genotypes, as indicated, measured by qRT-PCR. RNA was extracted from 5-week-old plants and *PR1* expression normalized to *UBQ10*. Standard deviation of three technical replicates is shown. C. Immunoblot analysis of total protein extracts from 5-week-old plants of the indicated genotypes separated by SDS-PAGE and probed with α-EDS1 antibodies. Ponceau S staining of the membrane is shown as loading control. The white line indicates where additional lanes from the same membrane were spliced out. D. *EDS1* transcript levels measured by qRT-PCR in the same genotypes and in the same material as in (B). E. Bacterial growth of *Pst* AvrRps4 in leaves of the indicated plant lines at 6 dpi. Five-week-old plants were spray-inoculated and bacterial titers determined. Error bars indicate standard errors of four biological replicates. Letters indicate statistically significant differences (ANOVA, Fisher’s LSD Post-hoc test, p < 0,05).

*A*. *thaliana* EDS1 forms resistance signaling complexes with either one of two sequence-related partners, PHYTOALEXIN DEFICIENT4 (PAD4) and SENESCENCE ASSOCIATED GENE101 (SAG101) [31, 40, 51, 52]. Whereas *PAD4* compensates genetically for a loss-of-function *sag101* mutation, *SAG101* only partially compensates for loss of *PAD4* in basal resistance against virulent pathogens and in TNL mediated ETI [40, 51, 53]. The enhanced disease susceptibility phenotype of a *pad4 sag101* double mutant is as penetrant as an *eds1* loss-of-function mutation and is not alleviated by over-expressing functional EDS1-HA [40, 51]. Thus, EDS1 requires PAD4 and, in the absence of PAD4, SAG101 for disease resistance signaling in basal immunity and ETI. We tested the genetic dependence of EDS1-YFP^NLS^ #A5 autoimmunity on *PAD4* and *SAG101* by crossing EDS1-YFP^NLS^ #A5 with Col *pad4-1* and *sag101-1* single null mutants or a Col *pad4-1 sag101-1* double mutant and selecting lines that were homozygous for the EDS1-YFP^NLS^ transgene and *eds1-2* in the respective homozygous mutant backgrounds. Developmental (Fig 4A) and *PR1* expression (Fig 4B) autoimmune phenotypes of EDS1-YFP^NLS^ #A5 were fully rescued by *pad4-1* and *pad4-1 sag101-1* but not by *sag101-1*. This indicates that autoimmunity caused by nuclear-enriched EDS1 has the same genetic requirements for *PAD4* and *SAG101* as *EDS1*-mediated basal immunity and ETI in wild-type plants. EDS1-YFP^NLS^ protein abundance was substantially lower in *pad4-1* and *pad4-1 sag101-1* mutant backgrounds, and similar to levels of native EDS1 in Col wild-type (Fig 4C). Reduced EDS1 accumulation can be largely attributed to reduced *EDS1* expression as measured by qRT-PCR in the same plants (Fig 4D). EDS1 is also directly stabilized by PAD4 or SAG101 [51, 52]. *A*. *thaliana* RRS1/RPS4 TNL resistance in EDS1-YFP^NLS^ #A5 against *Pst* AvrRps4 displayed the same genetic dependence on *PAD4* and *SAG101* as wild-type *EDS1* in Col (Fig 4E). We conclude that the defense-promoting actions of PAD4 or SAG101 predominantly target the EDS1 nuclear pool in plant immunity.

We next tested whether EDS1-YFP^NLS^ #A5 autoimmunity requires signaling by the defense hormone salicylic acid (SA) because EDS1-PAD4 promote SA-dependent and SA-independent defense pathways [41, 54–56]. Also, SA feeds-forward to induce *PAD4* expression [53]. For this, loss-of-function mutations in the SA biosynthetic enzyme gene *ISOCHORISMATE SYNTHESIS1* (*ICS1*, Col *sid2-1*) or the SA-response regulator gene *NON-EXPRESSOR OF PR GENES1* (*NPR1*, Col *npr1-1*) were introduced into the EDS1-YFP^NLS^ #A5 background. High accumulation of SA in 5-week-old EDS1-YFP^NLS^ #A5 was abolished in EDS1-YFP^NLS^ #A5/*sid2-1* plants (S2A Fig), confirming that SA in this line is produced mainly by ICS1 [57]. SA levels were not lower in EDS1-YFP^NLS^ #A5 /*npr1-1*, consistent with NPR1 operating downstream of SA accumulation [58]. Both *sid2-1* and *npr1-1* abolished enhanced expression of the SA-dependent *PR1* marker gene in EDS1-YFP^NLS^ #A5 (S2B Fig), but only slightly compromised accumulation of EDS-YFP^NLS^ protein (S2C Fig). Strikingly, neither *sid2-1* nor *npr1-1* suppressed EDS1-YFP^NLS^ #A5 stunting (S2D Fig). We concluded that EDS1-YFP^NLS^ #A5 immune-related growth defects are SA-independent or have a lower SA threshold.

Altogether, the genetic epistasis data suggest that EDS1-YFP^NLS^ autoimmunity operates by similar mechanisms as pathogen-elicited basal resistance or ETI, with EDS1-PAD4 controlled pathways branching into SA-dependent and SA-independent signaling sectors.

### Analysis of the EDS1-YFP^NLS^ #A5 transcriptome

**Fig 5:**
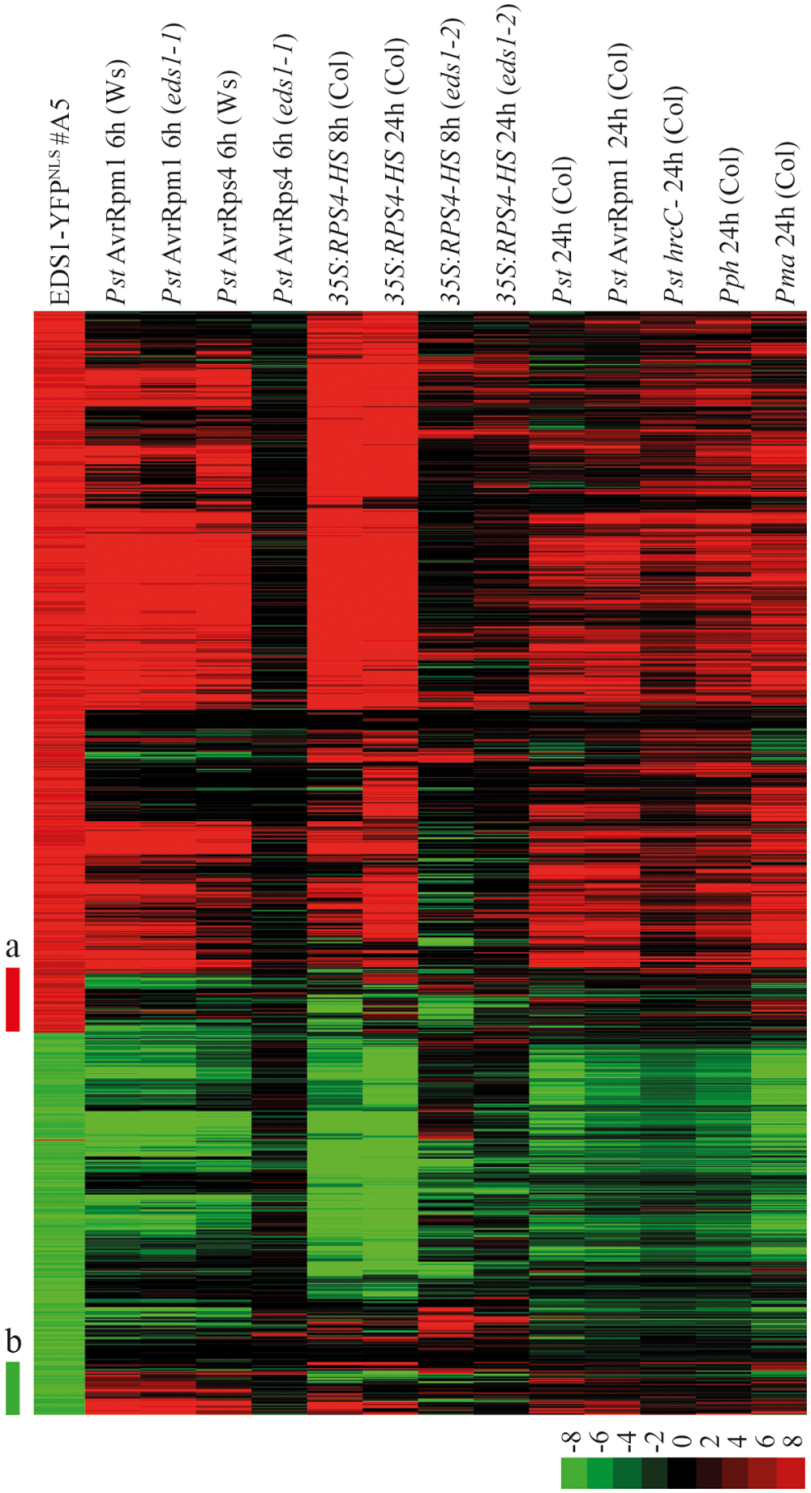
Transcriptome of EDS1-YFP^NLS^ #A5 transgenic plants. Transcript profiling was performed on ATH1 gene chips. Expression data for genes with ≥ 4-fold transcriptional changes in EDS1-YFP^NLS^ line #A5 compared to Col were taken for used for hierarchical clustering with transcriptome data sets from bacterial NLR-conditioned PTI and ETI responses, or 35S:RPS4-HS temperature-conditioned autoimmunity. Clusters of upregulated (a, red bar) and downregulated (b, green bar) unique for EDS1-YFP^NLS^ #A5 are marked. The color scheme represents fold-changes as indicated in the legend.

Previously, we found that shifting plants from high (28°C, permissive) to moderate (19°C, restrictive) temperature can be used to trigger *EDS1*-dependent autoimmunity in a transgenic *A*. *thaliana* RPS4 over-expression line (35S:RPS4-HS) [21]. Analysis of global gene expression changes in 35S:RPS4-HS and 35S:RPS4-HS *eds1-2* leaf tissues over a 24 h time course showed that temperature-conditioned RPS4 autoimmunity at 8 h and 24 h post temperature shift (pts) largely mirrors *EDS1*-dependent transcriptional reprogramming in *RRS1*/*RPS4* (TNL) ETI against *Pst* AvrRps4 [21]. Moreover, a set of *EDS1*-dependent induced or repressed marker genes from *Pst* AvrRps4-triggered tissues at 6 h post infection (hpi) displayed the same *EDS1*-dependent trends in 35S:RPS4-HS leaves at 8 h pts [21]. We performed Affymetrix ATH1 GeneChip analysis of 4-week-old untreated EDS1-YFP^NLS^ line #A5 and wild-type Col plants grown at 22°C to measure the extent to which EDS1-YFP^NLS^ #A5 autoimmunity resembles pathogen-elicited or temperature-induced *A*. *thaliana* immune responses. More than 2000 genes were significantly up- or down-regulated (p-value < 0.01, > 2-fold change) in EDS1-YFP^NLS^ line #A5 compared to Col at 22°C. Genes exhibiting at least 4-fold transcriptional differences in EDS1-YFP^NLS^ #A5 compared to Col were then used for hierarchical clustering with transcriptome data sets from bacterial NLR-conditioned PTI or ETI, as well as 35S:RPS4-HS temperature-conditioned autoimmunity (Fig 5 and S1 Table). This analysis established that the EDS1-YFP^NLS^ #A5 transcriptome was most similar to 35S:RPS4-HS gene expression changes at 8 h and 24 h pts and to those of ETI interactions (*Pst* AvrRps4, *Pst* AvrRpm1 6 h; Fig 5). The EDS1-YFP^NLS^ #5 transcriptome was most different to those of *Pst* AvrRps4-elicited or temperature-shift induced *eds1* mutant backgrounds (Fig 5). Notably, *EDS1*-dependent induced and repressed genes in the EDS1-YFP^NLS^ #A5 transcriptome overlapped substantially with *EDS1*-dependent induced and repressed gene sets in RRS1/RPS4-mediated ETI or 35S:RPS4-HS autoimmunity (Fig 5). Two clusters of induced and repressed genes were unique to EDS1-YFP^NLS^ #A5 (a and b in Fig 5, S2 Table) and might correspond to adaptation to prolonged defense activation in the EDS1-YFP^NLS^ #A5 line. The above results suggest that EDS1-YFP^NLS^ transgenic line #A5 behaves much like a TNL autoimmune background. Therefore, expressing high levels of nuclear targeted EDS1 is sufficient to induce transcriptional defense reprogramming without pathogen activation of a TNL receptor.

### Extragenic suppressors of EDS1-YFP^NLS^ #A3 seedling lethality

**Fig 6:**
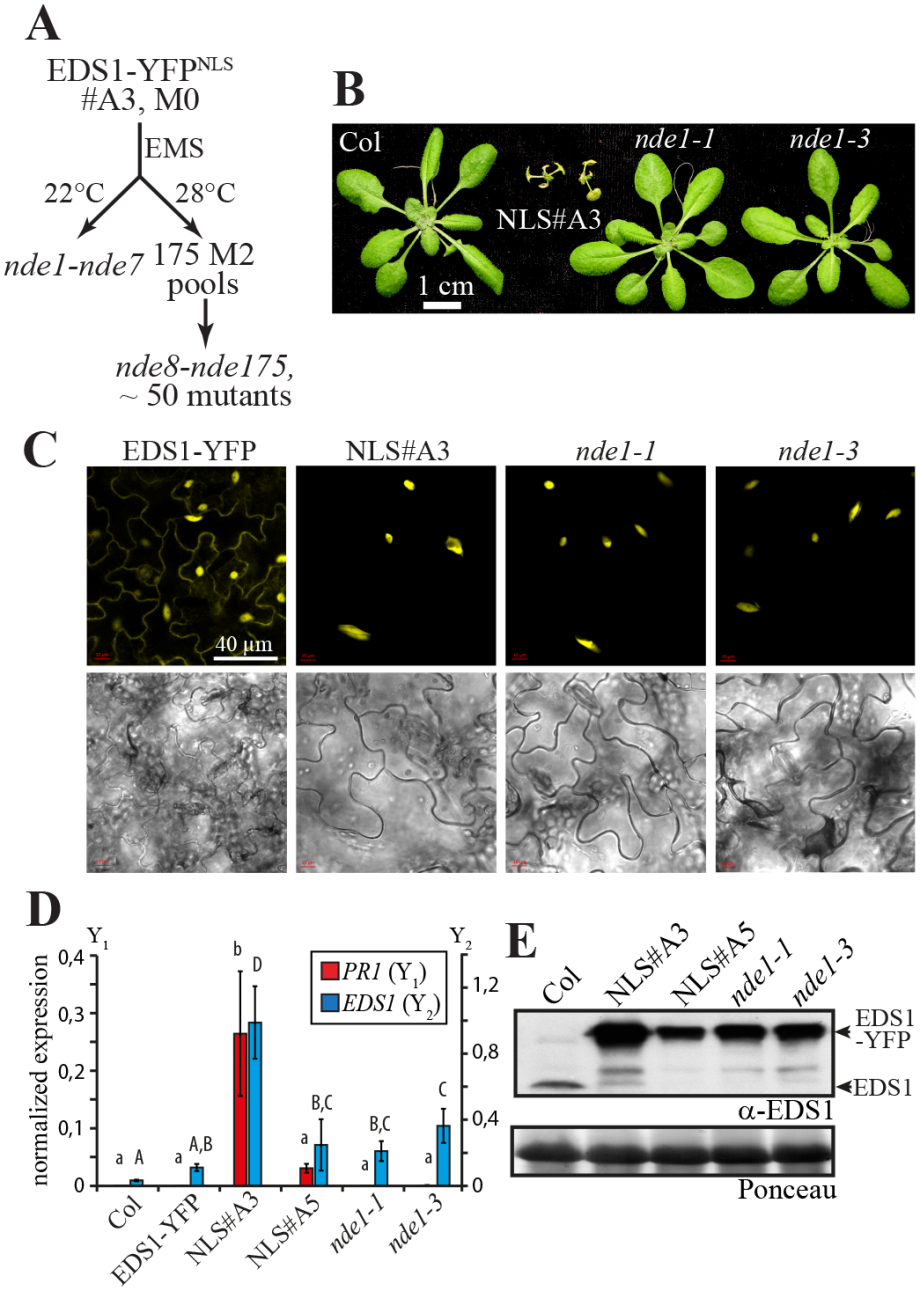
Isolation of *near death experience* (*nde*) extragenic suppressors of nuclear EDS1-induced autoimmunity. A. Screening scheme used for suppressor mutant isolation. The severe autoimmune line EDS1-YFP^NLS^ #A3 (NLS#A3) was used for EMS-mutagenesis. Mutants were obtained from screening both M_1_ plants and M_2_ pools under restrictive temperature conditions (22°C). B. Macroscopic growth phenotypes of 4-week-old *nde1* mutant plants (in the EDS1-YFP^NLS^ #A3 background) and control plants. C. Confocal live cell imaging of representative leaf epidermal cells from 4-week-old plants grown at 28°C shifted to 18°C for 24h. D. *PR1* (Y_1_ axis) and *EDS1* (Y_2_ axis) gene expression in the indicated lines measured by qRT-PCR. Plants were grown at 28°C and shifted to 18°C 24h before RNA extraction. Gene expression was normalized to *UBQ10*. Standard deviation of ≥ 3 biological replicates is shown. Letters indicate statistically significant differences (ANOVA, Fisher’s LSD Post-hoc test, p < 0,05). E. Immunoblot analysis of total protein extracts from the same plants as used in D.

We performed a genetic suppressor screen of the EDS1-YFP^NLS^ #A3 seedling lethality to identify components contributing to nuclear EDS1 autoimmunity. As shown above, high levels of EDS1-YFP^NLS^ expression in EDS1-YFP^NLS^ line #A3 caused rapid decline and eventual death of 3- to 4-week-old plants at moderate temperature (22°C) (Figs 1 and S1). The lethality phenotype was fully penetrant at 22°C and stable after three generations of propagating EDS1-YFP^NLS^ #A3 at 28°C. Seeds of EDS1-YFP^NLS^ line #A3 were mutagenized with ethyl methane sulfonate (EMS). This led to the isolation of mutants we have named ‘*n**ear* *d**eath* *e**xperience*’ (*nde*), which exhibited restored seedling viability and vigor to varying extents at 22°C. Seven putative dominant mutations (*nde1* to *nde7*) were identified by screening EMS mutagenized seedlings directly in the M_1_ generation (Fig 6A). A further 175 M_2_ pools (*nde8–175*; each derived from ~ 100 M_1_ plants propagated at 28°C) were screened at 22°C and ~ 50 additional *nde* mutants isolated from independent M_2_ pools (Fig 6A). Here, we describe analysis of a single *nde* complementation group containing alleles isolated in both the M_1_ (*nde1-1*, *nde1-3*) and M_2_ (*nde1-13*, *nde1-150* and *nde1-175*) suppressor screens.

*nde1-1* and *nde1-3* were initially scored as dominant suppressor mutations. When grown at 22°C, homozygous *nde1-1* and *nde1-3* M_3_ generation seedlings were indistinguishable from wild-type Col, whereas the parental EDS1-YFP^NLS^ line #A3 was severely stunted (Fig 6B). Further lowering of the growth temperature to 16°C did not produce *nde1-1* and *nde1-3* stunting or chlorosis. Homozygous *nde1-1* and *nde1-3* plants were backcrossed to the parental EDS1-YFP^NLS^ #A3 line and segregation of the seedling lethality phenotype at 22°C recorded in the F_2_ generation (BC1-F2). In both mutants, fully rescued *nde*, intermediate, and seedling lethal phenotypes segregated in a 1:2:1 ratio (*nde1-1*: 79:150:59, Chi^2^ = 3.28). This mode of inheritance suggests that *nde1-1* and *nde1-3* are loss-of-function alleles at single semi-dominant loci. EDS1-YFP^NLS^ localization remained entirely nuclear in *nde1-1* and *nde1-3* leaves, although YFP fluorescence intensity in the mutant lines was reduced compared to EDS1-YFP^NLS^ line #A3, assessed by confocal laser-scanning microscopy (Fig 6C). Therefore, we reasoned that phenotypic rescue was not due to interference with EDS1-YFP nuclear import but more likely reduced EDS1-YFP nuclear accumulation in *nde1-1* and *nde1-3*. The SA-response marker gene *PR1* was strongly induced in 3-week-old EDS1-YFP^NLS^ line #A3 seedlings shifted to 18°C for 24h, but not in *nde1-1* and *nde1-3* (Fig 6D). *EDS1* displayed a similar expression pattern to *PR1* in these seedlings (Fig 6D). Therefore, mutations in *nde1-1* and *nde1-3* attenuate *EDS1* mRNA accumulation under conditions inducing autoimmunity in the parental NLS#A3 line. Accumulation of EDS1-YFP^NLS^ protein was monitored in the same plants. EDS1 levels in *nde1-1* and *nde1-3* were lower than in the parental NLS#A3 line and comparable to those in line NLS#A5 showing autoimmunity under the same conditions (Fig 6E). Thus, suppression of autoimmunity in *nde1-1* and *nde1-3* is not solely caused by a reduction of EDS1-YFP^NLS^ levels.

The similarity of *nde1-1* and *nde1-3* phenotypes (Fig 6B) prompted us to perform an allelism test. *nde1-1* x *nde1-3* F_1_ plants grew normally at 22°C (S3A Fig). Approximately 400 F_2_ plants originating from four individual *nde1-1* x *nde1-3* F_1_ plants also showed no signs of stunting or chlorosis at 22°C (S3A Fig). Therefore, the possibility of F_1_ phenotypic rescue through actions of independent semi-dominant alleles (non-allelic non-complementation) was excluded, unless the independent non-allelic variants are closely linked. Segregation of a specific PCR marker for the *nde1-1* mutation generated after *A*. *thaliana* whole genome sequencing (see below) confirmed that *nde1-1* x *nde1-3* F_1_ plants were derived from true crosses (S3B Fig). The *nde1-13*, *nde1-150* and *nde1*-*175* mutations obtained in screens of EMS-mutagenized M_2_ plants fully rescued viability of EDS1-YFP^NLS^ #A3 at 22°C and were inherited in a semi-dominant manner. Also, *nde1-13*, *nde1-150* and *nde1*-*175* were found to be allelic with *nde1-1* after crossing and growing PCR-validated seedlings in the F_2_ generation. We concluded that *nde1-1*, *nde1-3*, *nde1-13*, *nde1-150* and *nde1-175* form a single complementation group of semi-dominant suppressors of nuclear EDS1 autoimmunity.

### Genetic interaction with a Landsberg-specific *RPP1-like* gene cluster underlies nuclear EDS1 autoimmunity

**Fig 7:**
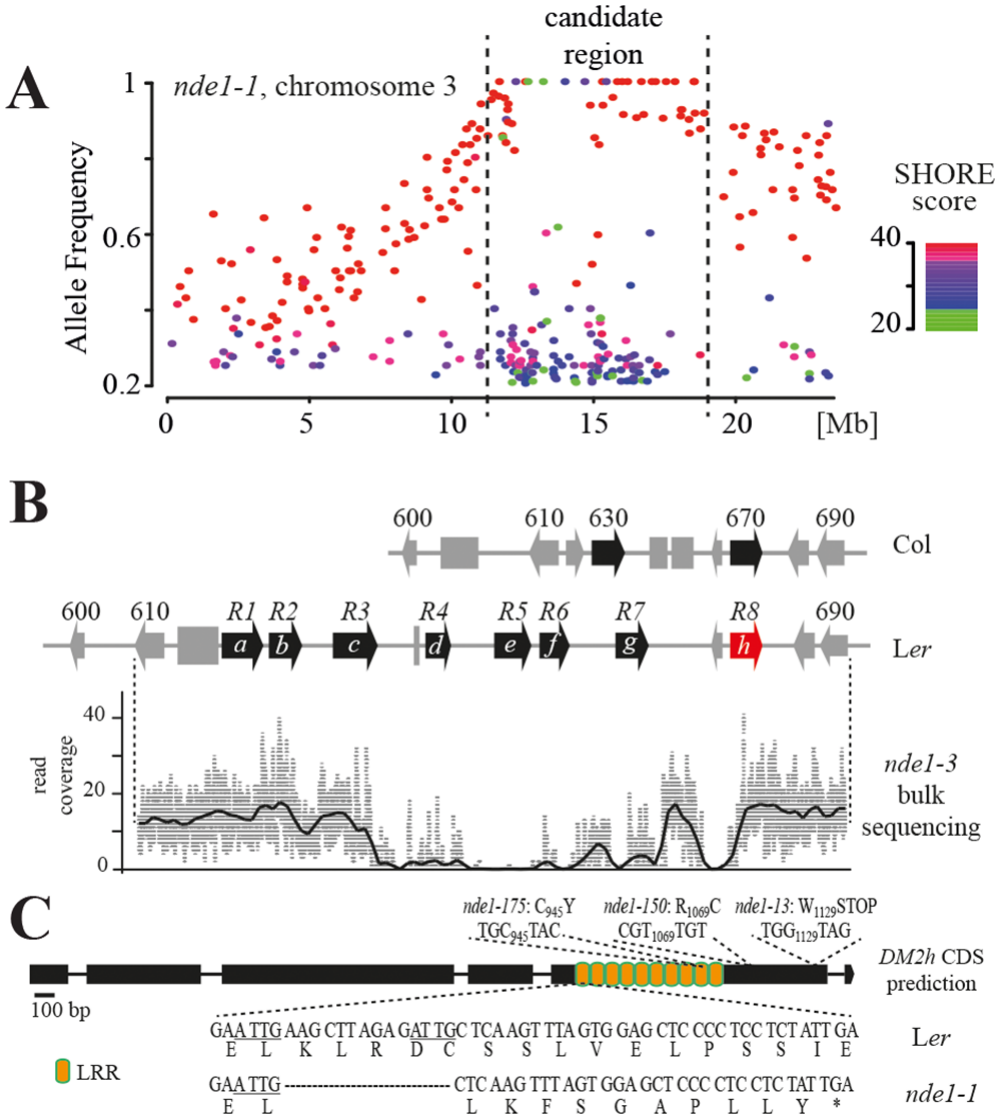
Mapping and identification of *NDE1*. A. Bulked segregant DNA from *nde1*-like BC1-F2 individuals and parental EDS1-YFP^NLS^ #A3 were Illumina sequenced. Allele frequency estimations of EMS changes on chromosome 3 in *nde1-1* bulked segregants after subtraction of SNPs from the parental line are shown. SHORE score is indicative of SNP quality, with higher scores corresponding to high confidence SNPs. B. Schematic representation of the *nde1*mapping interval and read coverage from *nde1-3* bulked segregant sequencing. The region in the Col reference genome is shown compared to accession L*er* [redrawn from 18]. NLR genes, non-NLR genes and transposable elements are indicated as black arrows, grey arrows and grey boxes, respectively. The *RPP1-like*^Ler^ *R8* (*DM2h*) gene affected in *nde1* alleles is highlighted in red. Numbers correspond to the last digits of At3g44XXX gene identifiers. *R1-R8* (*DM2a-h*) mark the *RPP1-like*^Ler^ genes, as described [27]. The read coverage curve was smoothened using running average and is drawn to scale with the genomic region from *A*. *thaliana* accession L*er*. C. Schematic representation of the CDS prediction for *RPP1-like*^Ler^ *DM2h* (*R8*) and lesions in different *nde1* alleles. The CDS was predicted from genomic DNA using fgenesh+ with RPP1-WsB [63] as protein input. A 14 bp deletion detected in *nde1-1* bulk segregant sequencing and point mutations detected in additional *nde1* alleles with consequences for the predicted DM2h (R8) protein are shown. DM2h-LRRs (S11 Fig) are indicated in orange.

We performed mapping-by-sequencing of the *nde1-1* and *nde1-3* mutations (see Materials and Methods) [59–61]. *A*. *thaliana* Col x L*er* SNPs were used to delineate the introgressed L*er* portion of DNA containing the *eds1*-2 mutation [41] to an approximately 6 Mb region in the parental EDS1-YFP^NLS^#A3 line (S4 Fig). Few polymorphisms with the Col reference sequence were detected in the remainder of the genome. Using SHOREmap [62], *nde1-1* and *nde1-3* were mapped to an approximately 5 Mb candidate region on the lower arm of chromosome 3, coinciding with the parental L*er* introgression (Figs 7A and S5). However, no locus containing a mutation in both *nde1-1* and *nde1-3* bulk sequences, expected for allelic mutations, was identified. We considered that *NDE1* might be a L*er*-specific gene or structural variant that is not present in the Col reference genome. Genetic crosses of EDS1-YFP^NLS^ #A3 and *nde1-1* to Col and Col *eds1-2*, respectively, confirmed that *NDE1* encodes a L*er*-specific autonecrosis-inducing factor which is lacking in Col (S3 Table).

*NDE1* was fine-mapped to a 90 kb interval in the Col reference genome by recombination mapping, and a physical contig of this region, which in accession L*er* spans 134 kb, assembled using a previous construction of the same locus in L*er* [27] (see Materials and Methods). Notably, the *NDE1* mapping interval contained QTL3^Ler^, a polymorphic region covering two TNL *RPP1-like* paralogs in Col [27]. The *RPP1-like* nomenclature derives from its close relatedness to a cluster of TNL *RPP1* genes in *A*. *thaliana* accession Ws-2 whose different paralogs confer isolate-specific *Hpa* (formally *Peronospora parasitica*) resistance [27, 63, 64]. In accession L*er*, the QTL3 region has expanded to contain seven complete and one truncated *RPP1-like* genes (denoted *R1-R8*, Fig 7B) [27] and the *RPP1-like*^Ler^ cluster was found to be the causal locus in a recessive deleterious epistatic interaction with *Strubbelig-Receptor Family 3 (SRF3)* allelic forms from *A*. *thaliana* accessions Kashmir (Kas-2) and Kondara (Kond-0), producing immune-related HI [25]. *RPP1*-like^Ler^ *R1-R8* correspond to *DM2a-h* paralogs of the *DANGEROUS MIX2* locus which underlies multiple negative epistatic interactions among *A*. *thaliana* genetic accessions leading to HI [18, 22]. For simplicity, we now refer to the *RPP1-like*^*Ler*^ *R1-R8* genes as *RPP1-like*^*Ler*^ *DM2a-h* (Fig 7B).

### Nuclear EDS1 autoimmunity requires *RPP1-like*^*Ler*^ *DM2h*

We reasoned that the *nde1* mutations might affect one or more of the *RPP1-like*^*Ler*^ *DM2a-h* (*R1*-*R8*) genes. Illumina reads from mapping-by-sequencing were re-analyzed against a reference genome containing the L*er NDE1* mapping interval. No canonical EMS changes were identified within the *NDE1* mapping interval but manual inspection revealed a prominent drop in read coverage along the *RPP1-like*^Ler^ cluster in *nde1-3*, extending from the *DM2c-d* (*R3-R4*) intergenic region to the *DM2h* (*R8*) 5’ region (Fig 7B). This was consistent with a large deletion or structural rearrangement in this line, which was confirmed by diagnostic PCR (S6A Fig). Similarly, a 14 bp deletion leading to a premature STOP was detected in the fifth exon of *DM2h* in *nde1-1* (Figs 7C and S6B). No additional SNPs were detected within the mapping interval in *nde1-1* bulk sequencing data, indicating that *NDE1* is *DM2h*. The *DM2h* coding region from *nde1-13*, *nde1-150* and *nde1-175* was therefore obtained by Sanger-sequencing. From this, EMS mutations leading to a premature stop in *nde1-13* (W_1129_Stop) or amino acid exchanges R_1069_C and C_945_Y, respectively in *nde1-150* and *nde1-175*, were detected (Fig 7C). Also, EDS1-YFP^NLS^ #A3 necrosis was restored in T_2_ progeny of the *nde1-1* mutant transformed with a *RPP1-like*^Ler^ genomic *DM2h* construct (S7 Fig). These results show that *DM2h* (*R8*) within the *RPP1-like*^Ler^ TNL gene cluster interacts genetically with EDS1-YFP^NLS^ resulting in autoimmunity.

Having identified *RPP1-like*^Ler^ *DM2h* as causal in nuclear EDS1 autoimmunity, we tested whether the EDS1-YFP^NLS^ #A3 or #A5 autoimmune response is accompanied by induced *DM2h* expression. *DM2h* expression was significantly reduced in *nde1* alleles compared to autoimmune lines EDS1-YFP^NLS^ #A5 and #A3, but there was only a two-fold increase in *DM2h* expression in the autoimmune lines (S8A Fig), although these had induced *PR1* expression (Fig 6D). This suggests that the *DM2h* gene itself is not strongly responsive to autoimmunity, in agreement with Alcazar et al (2014).

### *RPP1-like*^*Ler*^ *DM2h* is essential for *old3-1* and *SRF3*-induced autonecrosis

A previous screen for senescence-associated mutants in *A*. *thaliana* accession L*er* identified an EMS-induced mutation, *onset of leaf death 3–1* (*old3-1*) in the cysteine metabolic enzyme-coding locus *O-acetylserine(thiol)lyase A1*, which also displays negative epistasis with the *RPP1-like*^*Ler*^ gene cluster [65, 66]. Notably, *old3-1* caused autonecrosis in L*er*, but not Col, and was suppressed by amiRNA silencing of the *RPP1-like*^*Ler*^ cluster [65]. More specifically, silencing of *DM2g* (*R7*) most closely correlated with the suppression of *old3-1* dwarfism [65]. Here, we tested whether the *RPP1-like*^Ler^ *DM2h* gene contributes to autonecrosis induced by *old3-1*. From a Col x Col *eds1-2* cross, we selected two independent near isogenic lines (NILs) containing the *RPP1-like*^Ler^ locus and wild-type EDS1 from Col (Col-*RPP1-like*^Ler^). Similarly, we selected two independent NILs containing the *RPP1-like*^*nde1-1*^ locus and wild-type EDS1, but not the *EDS1-YFP*^*NLS*^ #A3 transgene from a Col x *nde1-1* cross (Col-*RPP1-like*^*nde1-1*^). Hence, the NILs differ mainly in the presence of a 14bp deletion in *DM2h* (*R8*) in Col *RPP1-like*^*nde1-1*^ but not Col-*RPP1-like*^Ler^. We used these NILs first to test whether *DM2h* (*R8*) contributes to other resistance responses not related to autoimmunity. NILs were infected with virulent (*Pst* DC3000, *Hpa* Noco2) and avirulent (*Pst* AvrRps4, *Hpa* Cala2) pathogen isolates (S9 Fig). There were no measurable differences in resistance between the NILs, suggesting that DM2h does not act as a helper NLR or generally lower NLR resistance thresholds.

**Fig 8:**
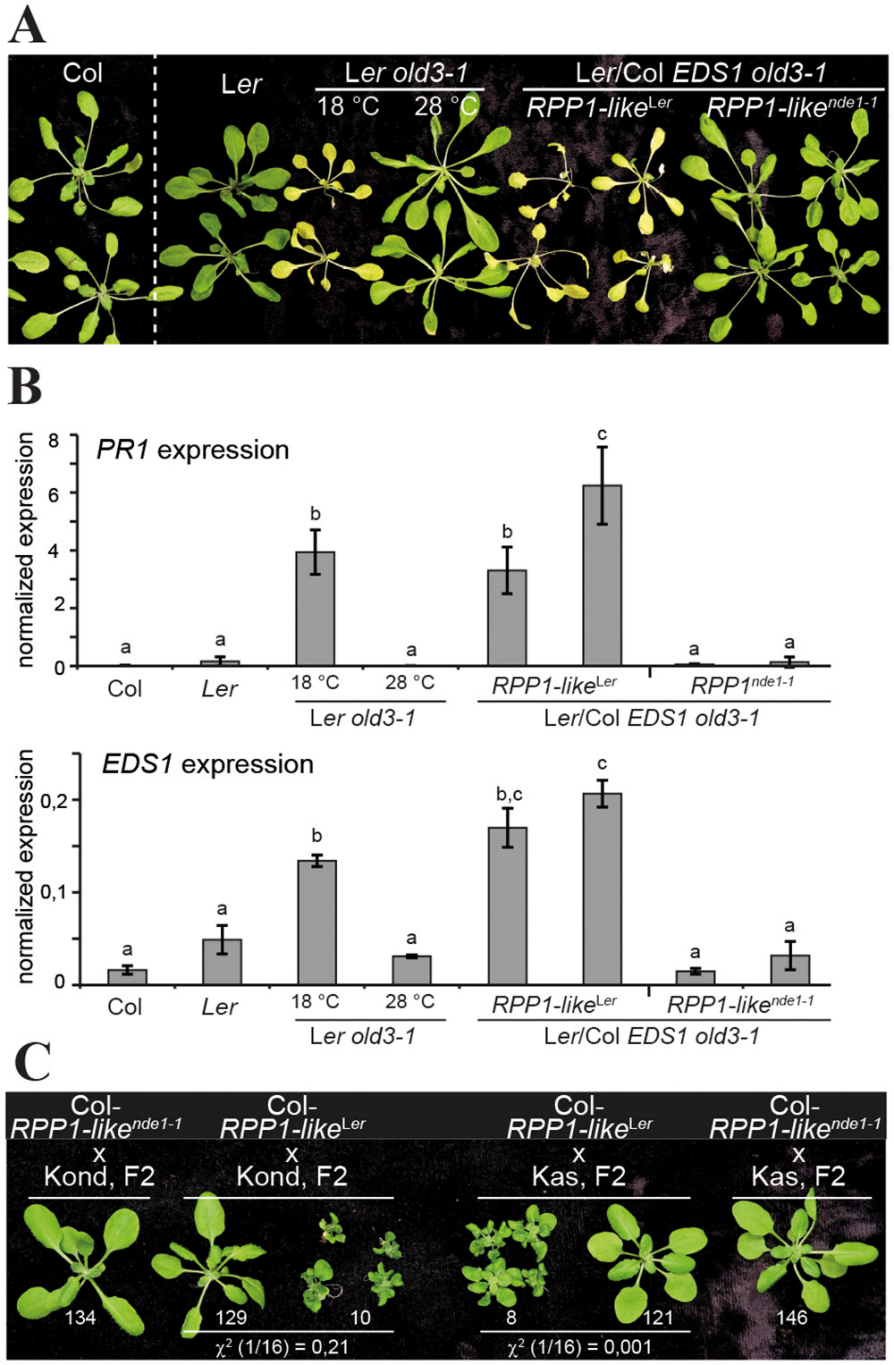
Contribution of *RPP1-like*^*Ler*^ *DM2h* to autoimmunity. A. Suppression of *old3-1*-induced autonecrosis by *nde1-1*. Col/Ler hybrids homozygous for *old3-1* and either *RPP1-like*^Ler^ or *RPP1-like*^*nde1-1*^ and control plants were grown at 28°C for three weeks. Plants were then shifted to 18°C or not (L*er old3-1* 28°C), and phenotypes recorded at 8 d pts. F_3_ families originating from crosses of *old3-1* with two independent Col-*RPP1-like*^Ler^ or Col-*RPP1-like*^*nde1-1*^ NILs were tested with similar results. Growth of Col plants was documented in the same experiment under the same conditions, but not in the same image, and is therefore separated by a dashed white line. B. *PR1* and *EDS1* gene expression in the lines shown in (A). RNA was extracted from the same plants at 24 h pts and cDNA used for transcript analysis by qRT-PCR. Gene expression was normalized to *UBQ10*. Standard deviation of three biological replicates is shown. Letters indicate statistically significant differences (ANOVA, Tukey’s Post-hoc test, p < 0,01). C. Suppression of *SRF3*^Kas/Kond^-induced hybrid necrosis by *nde1-1*. Col NILs containing either *RPP1-like*^Ler^ or *RPP1-like*^*nde1-1*^ were crossed to Kas and Kond, and occurrence of incompatible hybrids was recorded in 5-week-old F_2_ plants grown at 14°C (the restrictive temperature for Ler x Kas-2/Kond HI [27]. Numbers of wild type-like or necrotic plants observed in different F_2_ populations are indicated. Chi^2^ values for the segregation hypothesis of 1/16 necrotic/wild-type plants are indicated for segregating F_2_s.

The NILs developed normally and were crossed with L*er old3-1*. F_2_ plants homozygous for *old3-1* and either *RPP1-like*^Ler^ or *RPP1-like*^*nde1-1*^ were selected and symptoms of autonecrosis monitored in F_3_ progeny. *old3-1* plants grown at 28°C were not autonecrotic (Fig 8A) [65]. At 18°C, Col and L*er* were healthy but *old3-1* plants became necrotic (Fig 8A). Col/L*er* hybrids containing *old3-1* and *RPP1-like*^Ler^, but not hybrids containing *old3-1* and *RPP1-like*^*nde1-1*^ (lacking functional *DM2h*), also became necrotic (Fig 8A). Similarly, *PR1* and *EDS1* expression was upregulated in L*er old3-1* and Col/L*er RPP1-like*^Ler^ *old3-1* plants, but not Col/L*er RPP1-like*^*nde1-1*^ *old3-1*, *old3-1* grown at 28°C, or wild-type plants (Fig 8B). Induction of EDS1 in autoimmune lines was also detectable on western blots (S10A Fig). Dampening of *old3-1*-induced autonecrosis by the *nde1-1* mutation in *RPP1-like*^Ler^ *DM2h* was observed when isogenic F_1_ plants heterozygous for *old3-1/OLD3* and either homozygous for *RPP1-like*^Ler^ or heterozygous for *RPP1-like*^Ler^/*RPP1-like*^*nde1-1*^ were scored for necrosis (S10 Fig). We concluded from these genetic data that functional *RPP1-like*^Ler^ *DM2h* is essential for *old3-1* autoimmunity.

In a previous study, the *RPP1-like*^Ler^ *DM2c* (*R3*) gene was induced and contributed to negative epistasis between the *RPP1-like*^Ler^ cluster and *SRF3*^Kas/Kond^ in temperature-conditioned HI [67]. *DM2c* was also upregulated in the EDS1-YFP^NLS^ #A3 and #A5 autoimmune lines compared to EDS1-YFP (S8B Fig). Genetic analysis suggested that natively expressed *RPP1-like*^*Ler*^ *DM2c* was necessary but not sufficient for L*er* x Kas-2 autoimmunity [67]. We tested for a genetic contribution of *RPP1-like*^Ler^ *DM2h* to *SRF3*^Kas/Kond^ autoimmunity by analyzing F_2_ progeny from crosses of the Col-*RPP1-like*^Ler^ or Col-*RPP1-like*^*nde1-1*^ NILs with Kas-2 or Kond and scoring for incompatible hybrids (Fig 8C). In accordance with the previously described recessive genetic interaction between the *RPP1-like*^Ler^ locus and *SRF3*^Kas/Kond^ [25, 27], HI segregated at a 1/16 ratio in F_2_ progeny from crosses of *RPP1-like*^Ler^ introgression lines with Kas-2 or Kond (Fig 8C). By contrast, no incompatible hybrids emerged from crosses of *RPP1-like*^*nde1-1*^ with Kas-2 or Kond (Fig 8C). We concluded that *RPP1-like*^Ler^ *DM2h* is required for conditioning *SRF3*^Kas/Kond^ HI. Together, the data show that three different deleterious genetic interactions involve the *DM2h* gene of the *RPP1-like*^Ler^ TNL complex locus in *A*. *thaliana*.

## Discussion

In plants, TNL receptors recognizing different pathogen effectors converge on the nucleocytoplasmic regulator EDS1 to transcriptionally reprogram cells for ETI. Here, we find that low levels of *A*. *thaliana* EDS1 enriched in the nuclear compartment (in EDS1-YFP^NLS^ line #B2) are sufficient to confer basal immunity and TNL-triggered ETI against oomycete (*Hpa*) or bacterial (*Pst* DC3000) pathogen strains (Fig 2). Therefore, a small nuclear EDS1 pool appears to be competent in disease resistance signaling. In an earlier study we proposed a positive cytoplasmic role for EDS1 in *A*. *thaliana* immunity, based on intermediate resistance phenotypes of lines in which EDS1-YFP was mis-localized to the cytoplasm [38]. A reinterpretation of those data is that residual low amounts of nuclear EDS1 after subcellular mis-localization can confer resistance, at least with respect to the pathogen strains tested here. Nuclear-targeted EDS1 has identical genetic requirements for its signaling partner genes *PAD4* and *SAG101* as that of native, nucleocytoplasmic EDS1 in *A*. *thaliana* wild-type basal and TNL immunity (Fig 4). This highlights nuclear actions of PAD4 (or SAG101 when PAD4 is not present) in promoting EDS1 resistance, probably as EDS1 heteromeric complexes [40, 52]. We find that increased levels of *A*. *thaliana* nuclear-enriched EDS1 lead to autoimmunity exhibiting characteristic temperature-conditioned defense gene expression, accumulation of SA, and stunting of plant growth (in EDS1-YFP^NLS^ line #A5) or lethality (in EDS1-YFP^NLS^ line #A3) (Figs 1, S1, 2 and 5). Thus, above a certain threshold, nuclear EDS1 produces many of the hallmarks of TNL autoimmunity. In an extensive genetic screen for suppressors of the autoimmune response in EDS1-YFP^NLS^ line #3, we identify as causal four independent (*nde1*) mutations in one gene, *DM2h* (*R8*) within the *RPP1-like*^Ler^ TNL complex locus (Figs 6 and 7) [18, 22, 27]. Further genetic analysis shows that *RPP1-like*^Ler^ *DM2h* underlies two additional cases of *A*. *thaliana* autoimmunity, one with a mutated form of a cysteine metabolic enzyme (*O-acetylserine(thiol)lyase A1*) gene in *old3-1* within L*er* [65, 66], the other causing HI with allelic forms of a *receptor-like kinase* gene, *SRF3*, present in *A*. *thaliana* Kas-2 and Kond and other Central Asian accessions (Fig 8) [25, 27]. These data show that three different deleterious genetic interactions involving the *RPP1-like*^Ler^ gene cluster converge on *DM2h*.

Because *RPP1-like*^*Ler*^ *DM2h* (*R8*) is necessary for nuclear EDS1-PAD4 autoimmunity and defense gene expression (Figs 6, 7 and 8), we conclude that the DM2h protein directly or indirectly drives EDS1-PAD4 defense amplification. In one model, DM2h behaves as a weakly autoactive TNL protein which, in its native L*er* background or in the NILs expressing wild-type EDS1 (Figs 8 and S9), is effectively constrained. In other genetic backgrounds, DM2h weak autoactivity can be exposed in a temperature-dependent manner, as in incompatible hybrids (Fig 8). In this model, DM2h initiates EDS1/PAD4 signaling and DM2h autoactivity becomes deleterious when EDS1 nuclear accumulation rises above a threshold, producing autoimmunity.

An alternative explanation for dependence of EDS1-YFP^NLS^ line #A3 autoimmunity on *RPP1-like*^*Ler*^ *DM2h* is that EDS1 nuclear over-accumulation causes transcriptional mis-regulation of the *DM2h* gene as part of a feed-forward expression loop. However, *DM2h* expression was not significantly induced in EDS1-YFP^NLS^ lines #A3 or #A5 compared to non-autoimmune EDS1-YFP containing *RPP1*^Ler^. Similarly, *DM2c* was only mildly up-regulated in autoimmune EDS1-YFP^NLS^ lines (S8 Fig). In a previous analysis to identify genes within the *RPP1-like*^Ler^ locus underlying HI with *SRF3*^Kas/Kond^, strong up-regulation of *DM2c*^Ler^ (*R3*) but not *DM2h*^Ler^ (*R8*) correlated with autoimmunity [67]. In that study, *DM2h*^Ler^ was excluded as a causal gene based on the autoimmune phenotypes of selective *amiRNA* knock-down lines. Nevertheless, no single gene within the *RPP1-like*^Ler^ locus was able to reconstitute temperature-conditioned HI with *SRF3*^Kas/Kond^, leading the authors to propose co-actions of *DM2c*^Ler^ (*R3*) with one or more *RPP1-like*^Ler^ genes in the genetic incompatibility [67]. Our mapping of four independent *nde1* mutant alleles (*nde1-1*, *nde1-13*, *nde1-150*, *nde1-175*) to the *DM2h*^Ler^ gene, and establishing that *nde1-1* also suppresses HI in crosses with *A*. *thaliana* strains Kas-2 or Kond, provides genetic proof that *DM2h*^Ler^ (*R8*) is a key factor in nuclear EDS1 autoimmunity and Ler x Kas-2/Kond immune-related HI. Interestingly, DM2h (R8) contains a predicted N-myristoylation motif and a bipartite NLS (S11 Fig). The autoimmunity-inducing genetic interactors SRF3^Kas/Kond^ and EDS1-YFP^NLS^ localize to the plasma membrane [25] and nucleus (Fig 1C), respectively. Further analysis is needed to determine whether DM2h activity involves its membrane-tethering and/or nuclear localization.

It is significant that high levels of nucleocytoplasmic EDS1 expressed under its native or a constitutive (Cauliflower Mosaic virus 35S) promoter do not lead to autoimmunity [this study, 38, 40], unlike nuclear-targeted EDS1. We deduce from this that EDS1 nucleocytoplasmic trafficking through nuclear pore complexes [38] limits potentially hazardous actions of EDS1 in nuclear resistance signaling. This is likely to be important for maintaining cellular homeostasis and the trade-off between defense and fitness, especially under conditions when the plant is not being attacked by pathogens. Fusing EDS1 to a strong NLS might prolong transcriptional reprogramming activity of a nuclear EDS1 pool or draw other components into the nucleus. Because the EDS1-YFP^NLS^ #A5 plants do not show macroscopic defects until 4–5 weeks after planting at 22°C, EDS1-YFP^NLS^ is unlikely to cause severe clogging of the NPC import/export machinery which would have immediate effects on physiology and development. Artificially raising the EDS1 nuclear pool likely exposes ‘dangerous’ TNL alleles such as DM2h and drive cells and tissues into an activated immune response without a pathogen trigger. Here, DM2h immune reactivity does not appear to enhance ETI conditioned by other TNL genes (S9 Fig), suggesting a degree of specificity in *DM2h* co-action with *DM2c* and *EDS1*. Further analysis is required to establish whether *DM2h* and *DM2c* interact genetically or molecularly with each other, as found for a number of functional NLR and NOD-LRR receptor pairs [3, 5], or indeed with nuclear EDS1, in the different autoimmunity backgrounds. Whatever the mechanism of resistance deregulation, *DM2* locus steering of EDS1-YFP^NLS^ #A5 plants towards defense at the expense of growth involves SA-dependent and SA-independent signaling sectors (Figs 5 and S2), broadly resembling defense pathway bifurcations in pathogen-triggered EDS1-PAD4 basal and TNL immune responses [41, 54, 55].

Notably, stunting of EDS1-YFP^NLS^ line #A5 at moderate temperature was not alleviated by mutations in SA biosynthesis (*sid2-1*) or SA signaling (*npr1-1*) genes. By contrast, growth defects and necrosis, respectively, in a moderately incompatible L*er* x Kas-2 recombinant inbred line (RIL) and a severely dwarf *RPP1-like*^Ler^ x Kas-2 NIL, were fully suppressed by *sid2-1* [27]. The varying penetrance of SA pathway mutants in these two *EDS1*-dependent autoimmune backgrounds suggests that the consequences of EDS1 over-accumulation in EDS1-YFP^NLS^ line #A5 versus *RPP1-like*^Ler^ x Kas-2 HI are not identical, possibly due to different genetic modifiers or pathway fine-tuning between nuclear and nucleocytoplasmic EDS1.

Immune-related incompatibilities in plants between natural genetic variants (HI) often involve highly variable NLR gene clusters [18]. A body of evidence suggests that HI can expose divergent evolutionary trajectories of immune receptor genes through genetic drift, coevolution or local adaptation [18, 23, 67]. The occurrence of HI in crosses between genetic backgrounds might also shape which immune receptor or receptor cofactor genes can be assembled in any one genome [18, 19, 23]. The *A*. *thaliana* polymorphic *RPP1-like DM2* locus is especially remarkable in that genes within it underlie multiple, independent epistatic interactions causing autoimmunity. Whereas the *DM2* region in *A*. *thaliana* Col reference strain and related species *Arabidopsis lyrata* consists of just two *RPP1-like* genes [18, 67], the locus has expanded to contain seven complete *RPP1-like* genes in *A*. *thaliana* L*er* (Fig 7) and eight in accessions Bla-1 and Uk-1 [18]. These three accessions produce different *DM2*-based incompatibilities suggestive of a locus undergoing rapid evolutionary change [9, 12, 18].

The signatures of host-pathogen co-evolutionary conflict are especially evident at *A*.*thaliana* polymorphic *RPP* gene clusters or allelic variants recognizing different isolates of the adapted downy mildew pathogen *Hpa* [10, 68, 69]. The originally mapped *RPP1* resistance locus in accessions Ws-2 and Nd-0 encodes TNL variants (namely RPP1-WsB and RPP1-NdA) that confer allele-specific recognition of *Hpa*-derived Atr1 effector proteins by direct effector binding at the TNL receptor C-terminal LRR domain, cooperating with the central NB-ARC activation domain [63, 64, 70, 71]. Although it is not known whether genes within the different *DM2* haplotypes recognize specific *Hpa* or other pathogen strains, the *DM2h* LRR domain has a signature of diversifying selection among *A*. *thaliana* accessions, suggestive of variation in pathogen effector recognition surfaces [18]. In our study, the *DM2h*^Ler^ *nde1-175* mutation causes a non-synonymous C945Y exchange in an LRR consensus sequence residue of LRR9 (Figs 7C and S11). The C/N residues of the LxxLxLxxN/CxxL consensus form hydrogen bonds with backbone carbonyl groups throughout the entire LRR solenoid fold [72, 73]. The *nde1-175* mutation might thus perturb the overall shape of the DM2h-LRR domain or the local arrangement of LRR9 and neighboring LRRs. An R1069C exchange in *DM2h*^Ler^ *nde1-150* lies within the C-terminal LRR-flanking region. A W1129^SToP^ mutation in *nde1-13* causing a truncation encompassing only 41 amino acids (S11 Fig), points to functional importance of this extreme C-terminal region.

Unexpectedly, deletion mutants at the *RPP1-like*^Ler^ *DM2* locus in *nde1-1* (14 bp) and *nde1-3* (~ 50 kb) (Fig 7) were obtained in the M_1_ generation screen of EMS-mutagenized EDS1-YFP^NLS^ line #A3. When these M_1_ plants were initially grown under restrictive temperature conditions, healthy young leaves emerged from necrotic rosettes suggesting that the lesions in *nde1-1* and *nde1-3* might not have originated in embryonic cells targeted by EMS treatment but from recombination events later in seedling development. A spontaneous recombination event within the Col *RPP5* locus was reported for the *bal* variant in which there is 55 kb duplication encompassing the *SNC1* gene [74]. In *nde1-1*, a 4 bp (ATTG) micro-syntenic sequence flanking the 14 bp deletion might have directed somatic homologous recombination (SHR) at this position (Fig 7C). Although the origin of these genetic lesions remains speculative, unequal crossing-over and illegitimate recombination events are known to create sequence and locus-size variation in *Resistance* gene clusters [8, 75]. Also, gene recombination rates were reported to increase with biotic stress [76–78]. It is conceivable that the *nde1-1* and *nde1-3* alleles represent snapshots in the evolution of a plant NLR gene locus. The origin of the *RPP1-like*^Ler^ *DM2a-h* haplotype was recently traced to a natural *A*. *thaliana* population in Gorzów Wielopolski, Poland [67]. Genetic analysis of plants within this wild population showed that the *RPP1-like*^Ler^ *DM2a-h* locus has been maintained in genetically different individuals over many generations [67]. Further study of the Gorzów population will allow an exploration of the genetic and ecological forces shaping the evolution of this interesting TNL complex locus [67].

## Materials and Methods

### Plant material and growth conditions

Wild-type *Arabidopsis thaliana* accessions used were Col-0 (Col) and Landsberg *erecta* (L*er*). Col *eds1-2* [41], *pad4-1* [79], *sag101-1*, *pad4-1 sag101-1* [51], *sid2-1* [57], *npr1-1* [80], L*er old3-1* [66] mutant lines and the EDS1-YFP transgenic line [38] are published. An SV40 NLS was introduced at the 3’ end of the mYFP open reading frame by PCR, and a binary vector containing a BASTA^R^ plant-selectable marker and a pEDS1:gEDS1-YFP^NLS^ expression cassette within the T-DNA borders was generated as described [38]. *pad4-1*, *sag101-1*, *sid2-1* and *npr1-1* mutations were introduced into EDS1-YFP^NLS^ line #A5 by crossing and selecting homozygous backgrounds using PCR-based gene-specific markers (S3 Table). Plants were grown in soil at a 20°C: 22°C night: day cycle (200μE m^2^ s^-1^) and 60% relative humidity. For suppression of autoimmunity, plants were germinated at 22°C for 7d, and then shifted to 26°C/28°C (night/day) with 10h illumination. For homogeneous and stringent autoimmunity induction, plants were grown at either 20°/22°C or 26°C/28°C, and were then shifted to 18°C.

### Disease resistance assays

Spray inoculation of 4- to 6-week-old plants with *Pseudomonas syringae* pv *tomato* (*Pst*) strain DC3000 or *Pst* DC3000 expressing the effector AvrRps4 (*Pst* AvrRps4) was performed with bacterial suspensions of 1x10^7^ colony forming units ml^-1^ as described [38]. Bacterial entry was routinely checked by determining *in planta* bacterial titers at 3 hpi, and was similar between all genotypes used in this study. Conidiospore suspensions of 4x10^4^ spores ml^-1^ were used for *Hyaloperonospora arabidopsidis* (*Hpa*) infections. Lactophenol trypan blue (TB) staining of *Hpa*- and mock-infected leaves and pathogen spore counts were as described previously [81]. Disease resistance assays were repeated independently at least three times with similar results.

### ATH1 microarray gene expression

Total RNA of three independent biological replicates from 4-week-old Col and EDS1-YFP^NLS^ #A5 leaf tissues was isolated with an RNeasy Plant Mini kit supplied with RNase-Free DNase set (Qiagen) according to the manufacturer´s instructions. RNA quality was assessed on a Bioanalyzer (Agilent). Biotinylated cRNA was prepared according to manufacturer’s instructions from 1 μg total RNA (MessageAmp II-Biotin Enhanced Kit; Ambion). After amplification and fragmentation, 12.5 μg of cRNA was hybridized for 16 h at 45°C to a GeneChip ATH1-121501 Genome Array. GeneChips were washed and stained with Fluidics Script FS450-004 in the Affymetrix Fluidics Station 450 and scanned using a GeneChip Scanner 3000 7G. The data were analyzed with Affymetrix GeneChip Operating Software version 1.4 using Affymetrix default analysis settings and global scaling as the normalization method. Probe signal values were subjected to the GeneChip robust multi-array average (GC-RMA) summarization algorithm [82] to obtain expression level values. The microarray data were submitted to Gene Expression Omnibus (accession number GSE65415). Results were analyzed by the following linear model using the lmFit function in the limma package in the R environment: log2 (expression level value) sample + replicate. The eBayes function in the limma package was used for variance shrinkage in calculation of p-values. The Storey’s q-values were calculated using the q-value function in the q-value package from the p-values [83]. 1045 genes with at least 4-fold changes and q-value < 0.01 in EDS1-YFP^NLS^ #A5 compared to Col were selected for the clustering analysis. Expression values for these 1045 genes were extracted from publicly available data sets and were used for the clustering analysis. Hierarchical clustering analysis was performed using Cluster 3.0 software [84] with uncentered Pearson correlation and complete linkage, and visualized by Treeview software [84].

### qRT-PCR analysis

Total RNA was extracted using RNeasy Plant Mini Kit or TRI Reagent (Ambion). Reverse transcriptase (RT) reactions were performed with 1–2 μg of total RNA using SuperScriptII^™^ (Invitrogen) or RevertAid (Thermo Scientific). RT reactions were diluted 1:5 and 2 μl used for qPCR reactions on a Bio-Rad iQ5 or CFX Connect Real Time-PCR Detection System with EvaGreen (Biotium) or AbsoluteBlue (Thermo Scientific) dyes. *UBQ10* (At4g05320) transcript levels were used as an internal reference in all samples. Primer efficiencies were between 90–110% for all oligos, and data was analyzed using dCt. Gene expression was evaluated in at least three independent experiments with similar results.

### Protein expression analysis, live cell imaging and SA quantification

Total protein extracts were prepared by grinding leaf tissues in liquid nitrogen. Samples were resuspended in 2x Laemmli loading buffer (0.5 w/v), boiled for 10 min and centrifuged to remove cell debris. Proteins were separated by SDS-PAGE and electro-blotted to nitrocellulose membranes for protein gel blot analysis. Equal loading was monitored by staining membranes with Ponceau S (Sigma-Aldrich). Anti-EDS1 [51], anti-GFP (Roche), anti-Histone H3 (Agrisera) and anti-cFBPase (Agrisera) antibodies and secondary antibodies coupled to AP or HRP (Sigma, GE Healthcare) were used for detection. For live cell imaging, *Arabidopsis* leaves were examined with a Zeiss LSM780 confocal laser-scanning microscope directly after removing the leaves from plants grown at the different temperature regimes. Quantification of free and total SA in leaf tissues was done as previously described [85]. Similar results were obtained in at least three independent experiments.

### Isogenic mapping, recombination mapping, NIL generation, *DM2h* (*R8*) sequencing, and *nde1-1* mutant complementation

For isogenic mapping, *nde1-1* and *nde1-3* mutant plants (genotype Col *eds1-2 pEDS1*:*EDS1-YFP*^*NLS*^ *nde1-1/-3*) were backcrossed to the parental transgenic line EDS1-YFP^NLS^#A3 and plants showing the *nde* phenotype were selected from segregating F_2_ populations. Leaf material from > 100 segregants was pooled and DNA extracted using a DNeasy Maxi DNA Kit (Qiagen). DNA pools and DNA from the parental line EDS1-YFP^NLS^#A3 were used for library construction, and ~ 30M 100 bp single end reads per sample were produced on an Illumina HiSeq2500 at the Max-Planck Genome Centre, Cologne and analyzed with the short read analysis pipeline SHORE [86]. High quality (SHORE score > 20) SNPs between *nde1-1/-3* and the parental line were used in SHOREmap *backcross*. SNPs with allele frequency estimates ≥ 0.8 were considered as significant, defining mapping intervals from 10–23 Mb on chromosome 3 for both mutations. SNPs in this region were analyzed for their effect on gene coding sequences using TAIR 10. *nde1-1* was further fine-mapped using DNAs from ~ 400 single phenotyped plants from the same BC1-F2 population. SNPs detected by Illumina sequencing were converted to CAPS or dCAPS markers, and a final mapping interval supported by several recombinants on each side was defined by markers at 16.118 Mb and 16.428 Mb in the Col reference genome. No more additional SNPs for marker generation were available in this interval. For further mapping, transgenic line EDS1-YFP^NLS^#A3 was outcrossed to Col. F_2_ plants containing the EDS1-YFP^NLS^ transgene were selected by BASTA resistance and individuals containing the compatible Col^*NDE1*^ region (no signs of autonecrosis) were phenotypically selected by shifting BASTA-resistant plants to 18°C for 10d. Under these conditions, the EDS1-YFP^NLS^ transgene and *NDE1* in the hemizygous states were sufficient to induce macroscopic growth phenotypes. The Col^*NDE1*^ region was mapped using ~ 400 plants with L*er*/Col markers. A final mapping interval of ~ 90 kb in the Col reference was defined by markers JS676/677 and JS678/679 at 16.164 and 16.253 Mb on chromosome 3, respectively. A physical contig from L*er* containing both markers was constructed using FJ446580.1 [27] and scaffold 1526 from a reference-guided L*er* assembly [87]. An updated reference containing this contig, but not the respective region from Col, was created. Illumina reads were mapped against this reference using a CLC Genomics Workbench to generate data for Fig 7B and 7C. Col NILs containing *RPP1-like*^Ler^ or *RPP1-like*^*nde1-1*^ were generated by crossing Col *eds1-2* x Col or Col x *nde1-1*, respectively. F_2_ individuals with a recombination event between Col *EDS1* and the *RPP1-like*^Ler^ cluster were selected using L*er*/Col markers JS663/664, JS655/656 and an *eds1-2/EDS1* marker. Absence of the EDS1-YFP^NLS^ insertion was tested by PCR using oligos JS661/328 and JS661/665. L*er old3-1* plants were pollinated from four individual lines homozygous for *RPP1-like*^Ler/nde1-1^ and hetero- or homozygous for *EDS1/eds1-2*. For Sanger sequencing of additional *nde1* alleles, *DM2h* (*R8*) from *nde1-13*, *-150*, *-175* was PCR-amplified with JS724/725, and the PCR product cloned into a compatible Golden Gate plasmid by BsaI cut/ligation [88]. Two independent clones per allele were sequenced, and each contained only a single mutation. The same *DM2h* amplicon was also cloned from L*er*, the resulting construct transformed into *Agrobacterium* strain GV3101 pMP90 and *nde1-1* mutant plants transformed by floral dip. Primary transformants were selected on media containing Kanamycin, transferred to soil and cultivated at 28°C. T_2_ seeds were first cultivated at 28°C and subsequently shifted to 18°C to monitor induction of autoimmunity. Oligonucleotides are listed in S4 Table.

### Biochemical fractionation

For total protein extracts, 100 mg of Arabidopsis leaves were homogenized in liquid nitrogen and 500 μl of SDS extraction buffer (4% w/v SDS, 100 mM Tris-HCl pH 7.6, 100 mM DTT, protease and phosphatase inhibitor cocktails) were added. Samples were boiled for 10 min, centrifuged at 12000 x g for 10 min, and the supernatant recovered. Nuclei were extracted from 2g fresh leaves using a previously described method [89]. Briefly, fresh leaves were chopped in 30 ml nuclear extraction buffer (2.0 M hexylene glycol (2-methyl-2,4-pentandiol), 20 mM PIPES/KOH pH7.0, 10 mM MgCl2 and 5 mM 2-mercaptoethanol, protease and phosphatase inhibitor cocktails), filtered through 5 layers of cheesecloth, and subjected onto a 30% / 80% percoll density gradient. After centrifugation at 2000 x g for 30 min, the layer between 30–80% percoll was collected, loaded on 30% percoll, and re-centrifuged at 2000 x g for 30 min. The pellet was collected, mixed with 100 μl SDS-extraction buffer, and boiled for 10 minutes. Samples were centrifuged (10000 x g, 10 min), and the supernatant was collected as the nuclear protein fraction. Protein concentrations were determined using Pierce 660 nm absorbance assay in presence of IDCR reagent following the manufacturer’s protocol (Thermo Fisher Scientific). Coomassie Brilliant Blue staining of membranes was used as loading control.

## Supporting Information

S1 Table Expression data used for hierarchical clustering. (XLSX)

S2 Table EDS1-YFP^NLS^-specific, regulated genes. (XLSX)

S3 Table Segregation data for genetic crosses. (DOCX)

S4 Table Oligonucleotides used in this study. (DOCX)

S1 Fig Macroscopic and molecular phenotypes of EDS1-YFP^NLS^ and EDS1-YFP^nls^ transgenic lines. A. Macroscopic growth phenotypes of EDS1-YFP^NLS^ lines #B2, #A5 and #A3 with control plants at 22°C and 28°C. B. Immunoblot analysis of total protein extracts from 4-week-old plants grown at 22°C before and 24h after infection with *Pst* AvrRps4 bacteria. C. Confocal live cell imaging of representative leaf epidermal cells. Plants were germinated at 22°C (7d), subsequently cultivated at 28°C (14d) and shifted to 18°C 24h prior to imaging. D. Macroscopic growth phenotype of EDS1-YFP^nls^* transgenic and control lines. Plants were grown at 23/21°C under short day conditions (4 weeks) and subsequently under long day conditions (2 weeks) in a greenhouse. E. *PR1* marker gene expression in the indicated genotypes at 22°C and 28°C, measured by qRT-PCR. RNA was extracted from 5-week-old plants, expression was normalized to *UBQ10*, and standard deviation of 3 technical replicates is shown. F. *PR1* marker gene expression in the indicated genotypes 3d after shift to 18°C, normalized to *UBQ10*. Standard deviation of 4 biological replicates is shown. Letters indicate statistically significant differences (ANOVA, Fisher’s LSD Post-hoc test, p < 0,05). G. Bacterial growth of *Pst* AvrRps4 bacteria at 3 dpi. Plants were germinated at 22°C (7 d), subsequently cultivated at 28°C (20 d) and spray-inoculated. Error bars indicate standard deviation of 8 biological replicates. Letters indicate statistically significant differences (ANOVA, Fisher’s LSD Post-hoc test, p < 0,05).
(PDF)

S2 Fig SA accumulation and signaling in nuclear EDS1-induced autoimmunity. A. Accumulation of free SA in 5-week-old plants of the indicated genotypes. Plants were shifted to 19°C 7 d prior to metabolite extraction. Standard deviation of 3 technical replicates is shown. B. *PR1* marker gene expression in the indicated genotypes, measured by qRT-PCR. RNA was extracted from 5-week-old plants and expression normalized to *UBQ10*. Standard deviation of 3 technical replicates is shown. C. Immunoblot analysis of total protein extracts from the indicated genotypes separated by SDS-PAGE and probed with α-EDS1 antibodies. Ponceau S staining is shown as a loading control. All signals were detected simultaneously on a single membrane but additional lanes were spliced out, as indicated by the separated panels. D. Macroscopic growth phenotypes of 5-week-old plants of the indicated genotypes grown at 22°C and shifted to 19°C 7 d prior to phenotyping.
(PDF)

S3 Fig Allelism of the *nde1-1* and *nde1-3* mutations. A. Representative macroscopic growth phenotypes of plants of the indicated genotypes at 22°C. The number of individual plants analyzed is indicated below. B. Genotyping of plants from (A) with a molecular marker that differentiates between the parental lines used for crossing.
(PDF)

S4 Fig Illumina-sequencing of EDS1-YFP^NLS^ line #A3. EDS1-YFP^NLS^ line #A3 was Illumina-sequenced > 30x coverage. Col/L*er* polymorphisms were visualized using SHOREmap to delineate the *eds1-2* introgression originating from accession L*er*.
(PDF)

S5 Fig Mapping by sequencing of *nde1-1* and *nde1-3*. BC1-F2 bulked segregant DNAs from *nde1-1* and *nde1-3* were Illumina-sequenced and allele frequency estimates at EMS changes displayed using SHOREmap *backcross* after subtraction of SNPs from the parental EDS1-YFP^NLS^ #A3 line.
(PDF)

S6 Fig PCR-based detection of *nde1-1* and *nde1-3* lesions. A. Amplicons within *RPP1-like*^Ler^ genes *DM2d*, *e*, *g* and *h* (*R4*, *R5*, *R7* and *R8*) were made using the indicated primer combinations (S4 Table) on four independent DNA preparations from *nde1-1* or *nde1-3*. Optimized annealing temperatures and non-saturating cycling conditions were used to avoid non-specific amplification from ortholog-encoding *DM2a-DM2h* (*R1-R8)* loci. B. Detection of *nde1-1* with CAPS markers. PCR amplicons were generated using the indicated primer combinations, digested with HindIII and resolved on agarose gels. Due to non-specific amplification from ortholog-encoding *DM2a-DM2g* (*R1-R7)* loci, some uncleaved PCR product is also visible in controls, but the wild type *DM2h* (*R8)*-specific cleavage product is never detected in *nde1-1*. Primer positions and the HindIII restriction polymorphism are indicated in a scheme. Expected HindIII fragments from wild-type and *nde1-1* DNA are shown next to agarose gel images.
(PDF)

S7 Fig Complementation of the *nde1-1* mutant. *nde1-1* mutant plants were transformed with a genomic fragment containing *DM2h* under control of its native regulatory sequences. T_2_ plants and control lines were germinated at 22°C (7d), transferred to 28°C (12d) and shifted to 18°C for 10d. In both transgenic lines, necrotic seedlings were observed and counted. Segregation ratios indicate complementation of the *nde1-1* mutant phenotype by single T-DNA insertions in both lines.
(PDF)

S8 Fig *DM2h* and *DM2c* transcript levels in autoimmune EDS1-YFP^NLS^ lines and suppressor mutants. *DM2h* (A) and *DM2c* (B) expression was measured by qRT-PCR on the same samples used in Fig 6D. Standard deviation of ≥ 3 biological replicates is shown. Letters indicate statistically significant differences (ANOVA, Fisher’s LSD Post-hoc test, p < 0,05).
(PDF)

S9 Fig Resistance responses in NILs containing *RPP1-like*^*Ler*^ or *RPP1-like*^*nde1-1*^. A. 2,5-week-old plants of the indicated genotypes were infected with *Hpa* Cala2 and first true leaves stained with Trypan Blue at 6 dpi. Representative micrographs of infection sites are shown. fh—free hyphae; TN—trailing necrosis; HR—hypersensitive response. B. As in A, but plants were infected with *Hpa* Noco2 and sporulation determined at 7 dpi. Error bars indicate standard deviation of four biological replicates. Asterisk indicates statistically significant difference to Col (Student’s t-test, p < 0,05). C. Bacterial growth of *Pst* DC3000 bacteria at 3 dpi on 5-week-old spray-infected plants of the indicated genotypes. Error bars indicate standard deviation of 8 biological replicates. Letters indicate statistically significant differences (ANOVA, Fisher’s LSD Post-hoc test, p < 0,01). D. Bacterial growth assays as done in C but plants were infected with *Pst* AvrRps4 bacteria.
(PDF)

S10 Fig Suppression of *old3-1*-induced autonecrosis by *RPP1-like*^*nde1-1*^ in isogenic F_1_ plants. A. Immunoblot analysis of total protein extracts from the indicated genotypes separated by SDS-PAGE and probed with α-EDS1 antibodies. Protein samples were prepared from the same plants at the same stage as in Fig 8B. B. Col-*RPP1-like*^Ler^ and Col-*RPP1-like*^*nde1-1*^ NILs were crossed to L*er old3-1* mutant plants, F_1_ plants and controls grown at 23°C and phenotypes documented after 3 weeks. Col and L*er* plants are healthy, but *old3-1* and EDS1-YFP^NLS^ #A3 plants are necrotic. F_1_ plants from crosses of *old3-1* with Col-*RPP1-like*^Ler^ NILs, which are heterozygous for *old3-1/OLD3* and homozygous for *RPP1*^Ler^, are also necrotic, although not as severely as *old3-1* control plants. Autonecrosis is further reduced in F_1_ plants from crosses of *old3-1* with Col-*RPP1*^*nde1-1*^, heterozygous both for *old3-1/OLD3* and *RPP1*^Ler^*/RPP1*^*nde1-1*^ and thus lacking one copy of functional *RPP1-like*^Ler^ *DM2h* (*R8)* compared to F_1_ plants from crosses with Col-*RPP1like*^Ler^. C. Genotyping of plants from (A). Primer combinations and loci queried by different genetic markers are indicated. Primer sequences are given in S4 Table.
(PDF)

S11 Fig *RPP1-like*^*Ler*^ *DM2H* (*R8*) predicted amino acid sequence and motifs. Amino acid sequence corresponds to the CDS prediction shown in Fig 7. The predicted TIR domain (SMART search) is underlined. LRRs were annotated according to [18] and refined manually. Consensus positions are aligned and marked in red. Additional features (predicted NLS and N-myristoylation motif, mutations in *nde1* alleles) are boxed and indicated.
(PDF)
