# Supplementary material for: Arabidopsis thaliana DM2h (R8) within the Landsberg RPP1-like Resistance Locus Underlies Three Different Cases of EDS1-Conditioned Autoimmunity
Source: PLoS Genet. 2016 Apr 15;12(4):e1005990. doi: 10.1371/journal.pgen.1005990 (PMC4833295; doi:10.1371/journal.pgen.1005990)
Supplement: S3 Table — (DOCX) [file pgen.1005990.s003.docx]

**Table S3**: Segregation of the EDS1-YFP^NLS^ #A3 autonecrosis phenotype in the F_2_ generation of different genetic crosses

|  | cross | WT-like  (compatible) | NLS#A3-like ^a^  (incompatible) | segregation  (hypothesis) | Chi^2^ |
| --- | --- | --- | --- | --- | --- |
| 1 | Col *eds1-2* x NLS#A3 | 70 | 169 | 1:3 ^b^ | 2.3 |
| 2 | Col x NLS#A3 | 110 | 134 | 1:3  7:9 ^c^ | 52.5  0.2 |
| 3 | Col *eds1-2* x *nde1* | 83 | 114 | 1:3  7:9 | 30.8  0.2 |
| 4 | NLS#A3 x *nde1* | 79 | 209 | 1:3 | 0.9 |
| 5 | Col x *nde1* | 372 | 0 | N/A | N/A |

^a^ Plants were shifted from 28^o^C to 18°C after 14d. All plants showing autonecrosis at 10d post temperature shift were considered as EDS1-YFP^NLS^ #A3-like; WT = wild-type, no signs of autonecrosis.

^b^ Induction of autonecrosis by EDS1-YFP^NLS^ #A3 transgene as single, dominant trait

^c^ Recessive epistasis of a second, unlinked locus suppressing autonecrosis

Genetic expectations:

1. 1:3 segregation of autonecrosis phenotype as a dominant trait caused by a single locus, the EDS1-YFP^NLS^ #A3 transgene, functioning dominantly in autoimmunity induction^a^.
2. 1:3 segregation if second locus necessary for autoimmunity induction (*NDE1*) is also present in Col. 7:9 segregation if second locus is only present in NLS#A3 (Col *eds1-2*) background. EDS1-YFP^NLS^ #A3 transgene and a second, unlinked locus (*NDE1*) both function dominantly in autoimmunity induction^a^.
3. 1:3 segregation for autoimmunity caused by a single, dominant locus. 7:9 segregation for autoimmunity depending on two unlinked loci (NLS#A3 transgene from *nde1-1*, *NDE1* from Col *eds1-2*). Both loci function dominantly^a^.
4. 1:3 segregation for suppression of autoimmunity depending on the homozygous *nde1-1* allele; NLS#A3 transgene does not segregate; control.
5. *NDE1* is not present in Col. No segregation of NLS#A3-like plants.
