# Supplementary material for: Arabidopsis thaliana DM2h (R8) within the Landsberg RPP1-like Resistance Locus Underlies Three Different Cases of EDS1-Conditioned Autoimmunity
Source: PLoS Genet. 2016 Apr 15;12(4):e1005990. doi: 10.1371/journal.pgen.1005990 (PMC4833295; doi:10.1371/journal.pgen.1005990)
Supplement: S4 Table — (DOCX) [file pgen.1005990.s004.docx]

Supplemental Table 3: Oligonucleotides used in this study

| name | sequence 5’->3’ | purpose |
| --- | --- | --- |
| NP_sid2_F | GCAGTCCGAAAGACGACCTCGAG | *sid2-1* detection, MfeI digestion |
| NP_sid2_R | CTATCGAATGATTCTAGAAGAAGC |  |
| NP_npr1_F | TGAGTGCGGTTCTACCTTCC | *npr1-1* detection, NlaIII digestion |
| NP_npr1_R | ATGTCTCGAATGTACATAAGG |  |
| AC98 | CAAACGTCAAGAGAGCTGAG | *eds1-2/EDS1/EDS1-YFP* genotyping |
| EDS6 | GTGGAAACCAAATTTGACATTAG |  |
| EDS105/E2 | ACACAAGGGTGATGCGAGACA |  |
| *PR1* fw | TTCTTCCCTCGAAAGCTCAA | *PR1* qPCR |
| *PR1* rev | AAGGCCCACCAGAGTGTATG |  |
| *UBIQ* fw | AGATCCAGGACAAGGAGGTATTC | *UBQ10* qPCR |
| *UBIQ* rev | CGCAGGACCAAGTGAAGAGTAG |  |
| *EDS1* fw | CGAAGACACAGGGCCGTA | *EDS1* qPCR |
| *EDS1* rev | AAGCATGATCCGCACTCG |  |
| *CBP60g* fw | GGCGAGAAGTGAAGCTTTTG | *CBP60g* qPCR |
| *CBP60g* rev | GCGAAAATCCTTGACGGTTA |  |
| *PBS3* fw | ACACCAGCCCTGATGAAGTC | *PBS3* qPCR |
| *PBS3* rev | CCCAAGTCTGTGACCCAGTT |  |
| *ICS1* fw | TTCTGGGCTCAAACACTAAAAC | *SID2* qPCR |
| *ICS1* rev | GGCGTCTTGAAATCTCCATC |  |
| *JS676* | TCACTTTCTCCGAACAACCA | L*er*/Col marker, CAPS XmnI, 16.164 Mb on chr. 3 |
| *JS677* | CCATCAACATCAGCACCAAC |  |
| *JS678* | ATGAACCCTCGTGAAGAAGCA | L*er*/Col marker, CAPS BglII, 16.253 Mb on chr. 3 |
| *JS679* | AGACCATTCGTATCCACACC |  |
| JS663 | CGGAATTGATGTTTTGGACC | Ler/Col marker, 15.985 Mb on chr. 3 |
| JS664 | TACATTCTACAACCATGTAGCC |  |
| *JS655* | CAAAAGAAATGCAACGAGAC | Ler/Col marker, 16.275 Mb on chr. 3 |
| *JS656* | tgggagagaatgaaatggtc |  |

| *JS661* | ATGTCTTGTCACAGTGCTCG | presence/absence of EDS1-YFP^NLS^#A3insertion |
| --- | --- | --- |
| *JS328* | TGGGTTTTTATGATTAGAGTCC |  |
| *JS665* | GCTTGAGAGATCGACGGAGA |  |
| *JS724* | tttggtctcaATTCGAAAGCCAGAGGCAGATAAGAAG | *DM2h/R8* amplicon |
| *JS725* | tttggtctcaAAGCGCAATCAAGCGCATTACAGATGG |  |
| *JS709* | AGTACAAGAAAATACTTTTTG | *DM2e/R5* amplicon |
| *JS710* | AGAACTTCTTTGCAAGAACCC |  |
| *JS601* | tttggtctcaggagTAGGCTTCTTCATATTTTTTAGAA | *DM2d/R4* amplicon |
| *JS602* | tttggtctcatcgtACTTGCTCTTTTGGTTTACCTCTA |  |
| *JS684* | tttggtctcaggagatcatgcacacatcaactcg | *DM2g/R7* amplicon |
| *JS685* | tttggtctcatcgtTAAATCTCAAGGTTGTACGAAG |  |
| *JS686* | tttggtctcatcgtGATCGACTTACTCCTCTCTA | *DM2h/R8* amplicon |
| *JS687* | tttggtctcaggagCCGCAGCTTTTACAAAATTCA |  |
| *JS719* | AGAATCGATGCTAGTTTTCAACC | *nde1-1* detection, CAPS HindIII |
| *JS714* | TTGAGTAATTCTGAAGACTTGA |  |
| *JS716* | TTCAAGATATAGTTCCTCAAGT |  |
| *JS720* | AGTTGATGTTGATTGGAAGCTCC |  |
| *JS433* | agttactcgaaaatgacatgatc | marker linked to *nde1-1* |
| *JS438* | gtaggtattgattatcatcagc |  |
| *pad4-1 marker* | GCGATGCATCAGAAGAG  TTAGCCCAAAAGCAAGTATC | *pad4-1* genotyping, BsmFI digestion |
| *sag101-1 marker* | ATGCAAGGAGGTCAAGATCG  GGTGCAGCAAAACCCACACTTTTACTTC | detection *sag101-1* insertion |
| *SAG101 marker* | ATGCAAGGAGGTCAAGATCG  TTGTGACTTACCATAACTCTCG | detection wt *SAG101* |
| *JS896_R3qRT* | TGGATTTACGTGAGGAAGAATTG | DM2c/R3 qRT  (Alcazar, 2014) |
| *JS897_R3qRT* | GAAGTGAAGCAGTTTCTGTCT |  |
| *JS898_R8qRT* | GCAACTAATCTCGAAGAATTG | DM2h/R8 qRT  (Alcazar, 2014) |
| *JS899_R8qRT* | AGACTTGAGCACCTTTGGAGATA |  |
