# Supplementary figures and images for: Arabidopsis thaliana DM2h (R8) within the Landsberg RPP1-like Resistance Locus Underlies Three Different Cases of EDS1-Conditioned Autoimmunity
Source: PLoS Genet. 2016 Apr 15;12(4):e1005990. doi: 10.1371/journal.pgen.1005990 (PMC4833295; doi:10.1371/journal.pgen.1005990)

Supplemental Figure S1

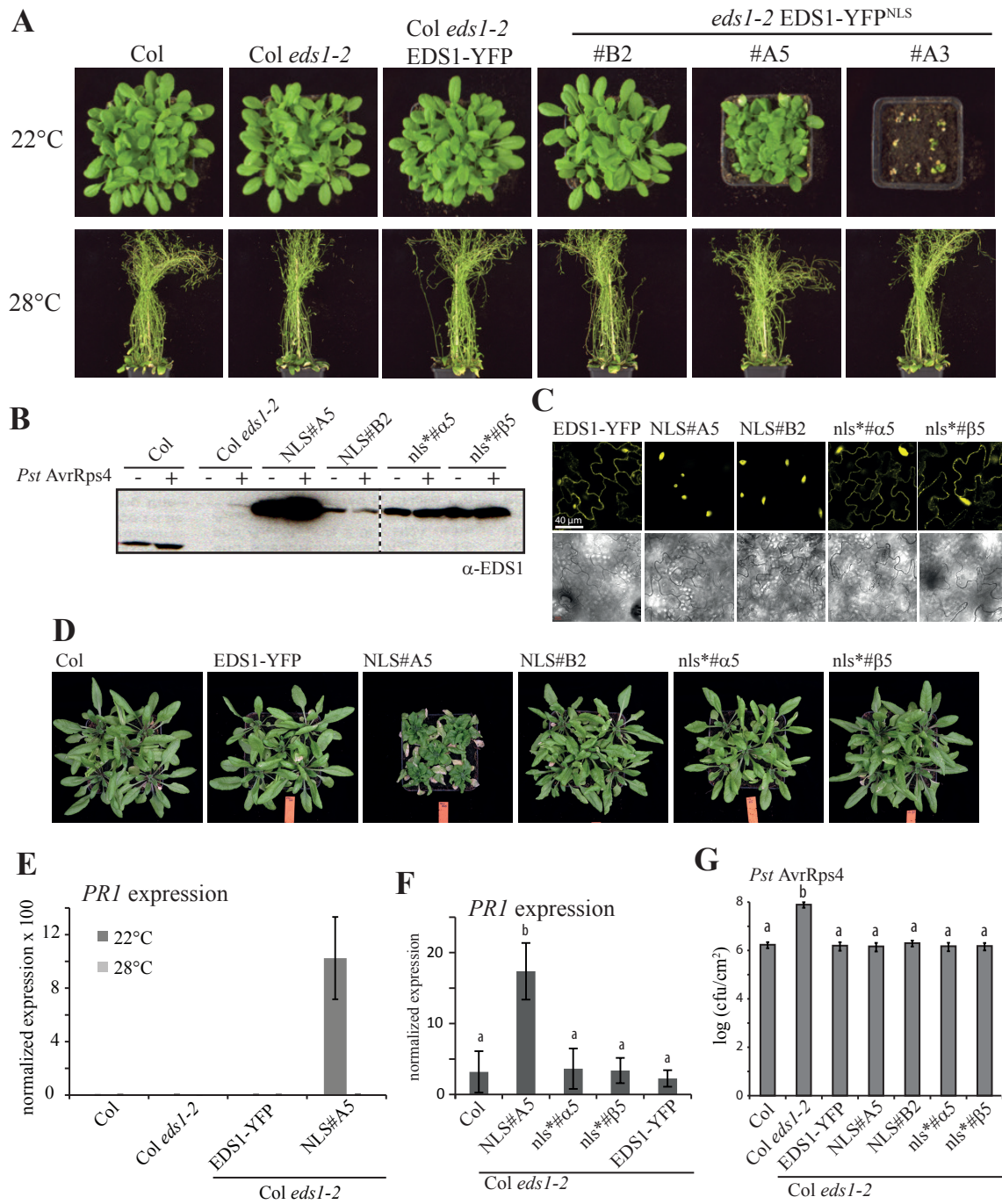

Supplement: S1 Fig — A. Macroscopic growth phenotypes of EDS1-YFPNLS lines #B2, #A5 and #A3 with control plants at 22°C and 28°C. B. Immunoblot analysis of total protein extracts from 4-week-old plants grown at 22°C before and 24h after infection with Pst AvrRps4 bacteria. C. Confocal live cell imaging of representative leaf epidermal cells. Plants were germinated at 22°C (7d), subsequently cultivated at 28°C (14d) and shifted to 18°C 24h prior to imaging. D. Macroscopic growth phenotype of EDS1-YFPnls* transgenic and control lines. Plants were grown at 23/21°C under short day conditions (4 weeks) and subsequently under long day conditions (2 weeks) in a greenhouse. E. PR1 marker gene expression in the indicated genotypes at 22°C and 28°C, measured by qRT-PCR. RNA was extracted from 5-week-old plants, expression was normalized to UBQ10, and standard deviation of 3 technical replicates is shown. F. PR1 marker gene expression in the indicated genotypes 3d after shift to 18°C, normalized to UBQ10. Standard deviation of 4 biological replicates is shown. Letters indicate statistically significant differences (ANOVA, Fisher’s LSD Post-hoc test, p < 0,05). G. Bacterial growth of Pst AvrRps4 bacteria at 3 dpi. Plants were germinated at 22°C (7 d), subsequently cultivated at 28°C (20 d) and spray-inoculated. Error bars indicate standard deviation of 8 biological replicates. Letters indicate statistically significant differences (ANOVA, Fisher’s LSD Post-hoc test, p < 0,05). (PDF) [file pgen.1005990.s005.pdf]

Supplemental Figure S2

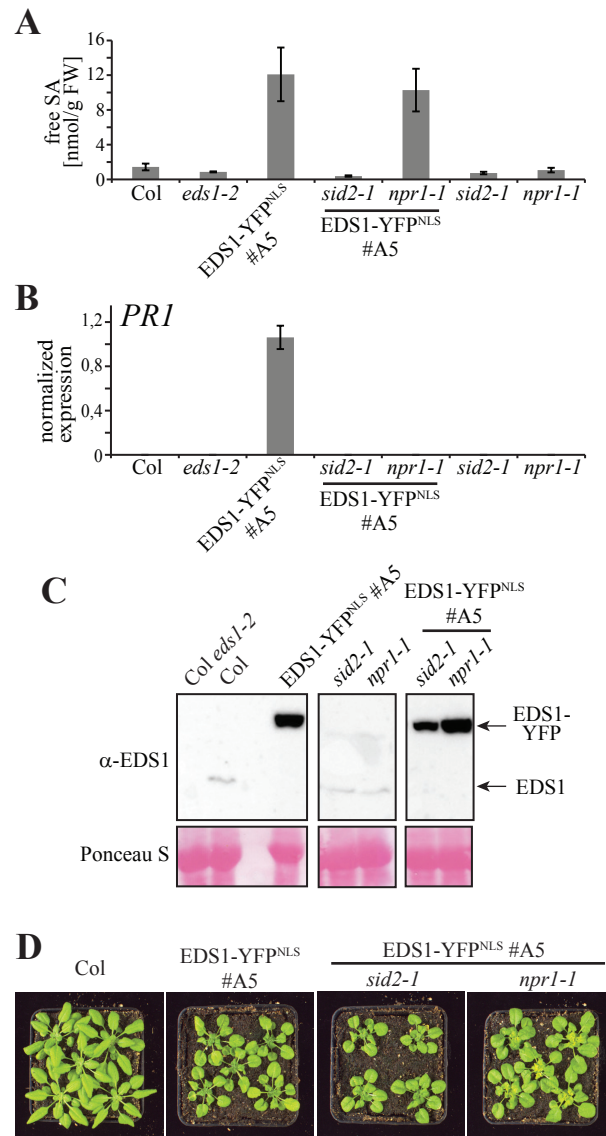

Supplement: S2 Fig — A. Accumulation of free SA in 5-week-old plants of the indicated genotypes. Plants were shifted to 19°C 7 d prior to metabolite extraction. Standard deviation of 3 technical replicates is shown. B. PR1 marker gene expression in the indicated genotypes, measured by qRT-PCR. RNA was extracted from 5-week-old plants and expression normalized to UBQ10. Standard deviation of 3 technical replicates is shown. C. Immunoblot analysis of total protein extracts from the indicated genotypes separated by SDS-PAGE and probed with α-EDS1 antibodies. Ponceau S staining is shown as a loading control. All signals were detected simultaneously on a single membrane but additional lanes were spliced out, as indicated by the separated panels. D. Macroscopic growth phenotypes of 5-week-old plants of the indicated genotypes grown at 22°C and shifted to 19°C 7 d prior to phenotyping. (PDF) [file pgen.1005990.s006.pdf]

# Supplemental Figure S3

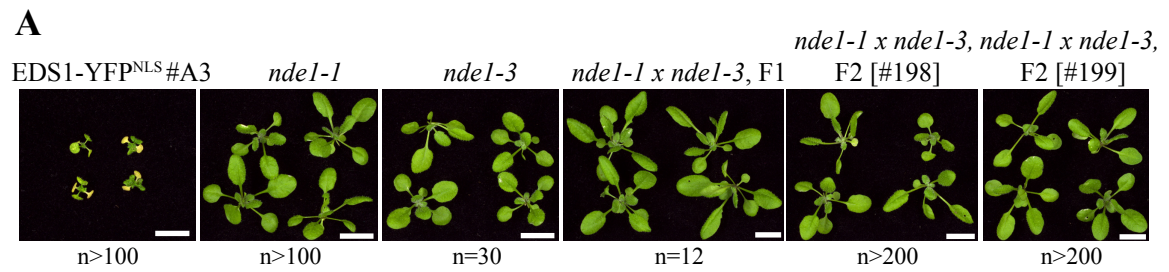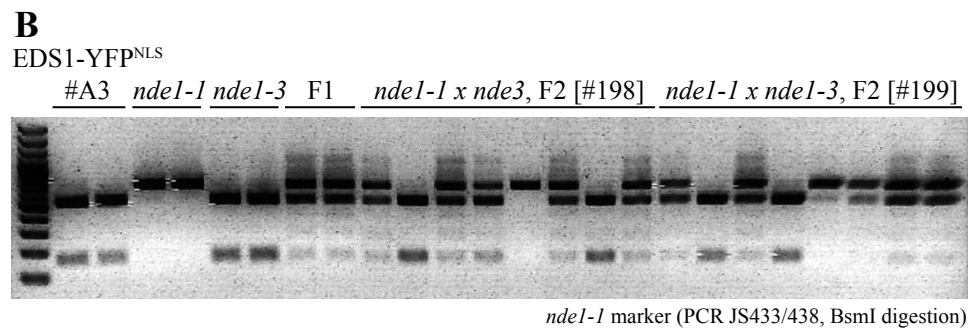

Supplement: S3 Fig — A. Representative macroscopic growth phenotypes of plants of the indicated genotypes at 22°C. The number of individual plants analyzed is indicated below. B. Genotyping of plants from (A) with a molecular marker that differentiates between the parental lines used for crossing. (PDF) [file pgen.1005990.s007.pdf]

Supplemental Figure S4

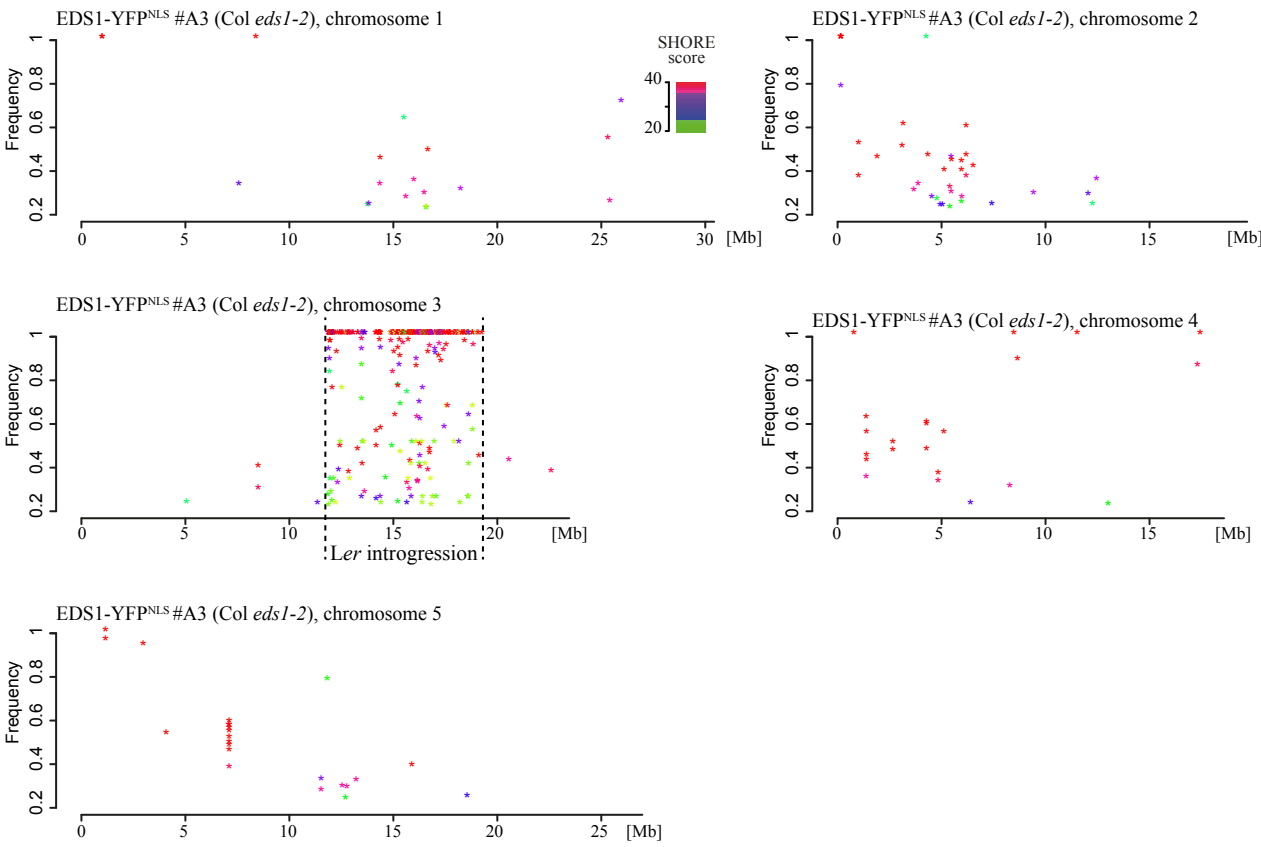

Supplement: S4 Fig — EDS1-YFPNLS line #A3 was Illumina-sequenced > 30x coverage. Col/Ler polymorphisms were visualized using SHOREmap to delineate the eds1-2 introgression originating from accession Ler. (PDF) [file pgen.1005990.s008.pdf]

Supplemental Figure S5

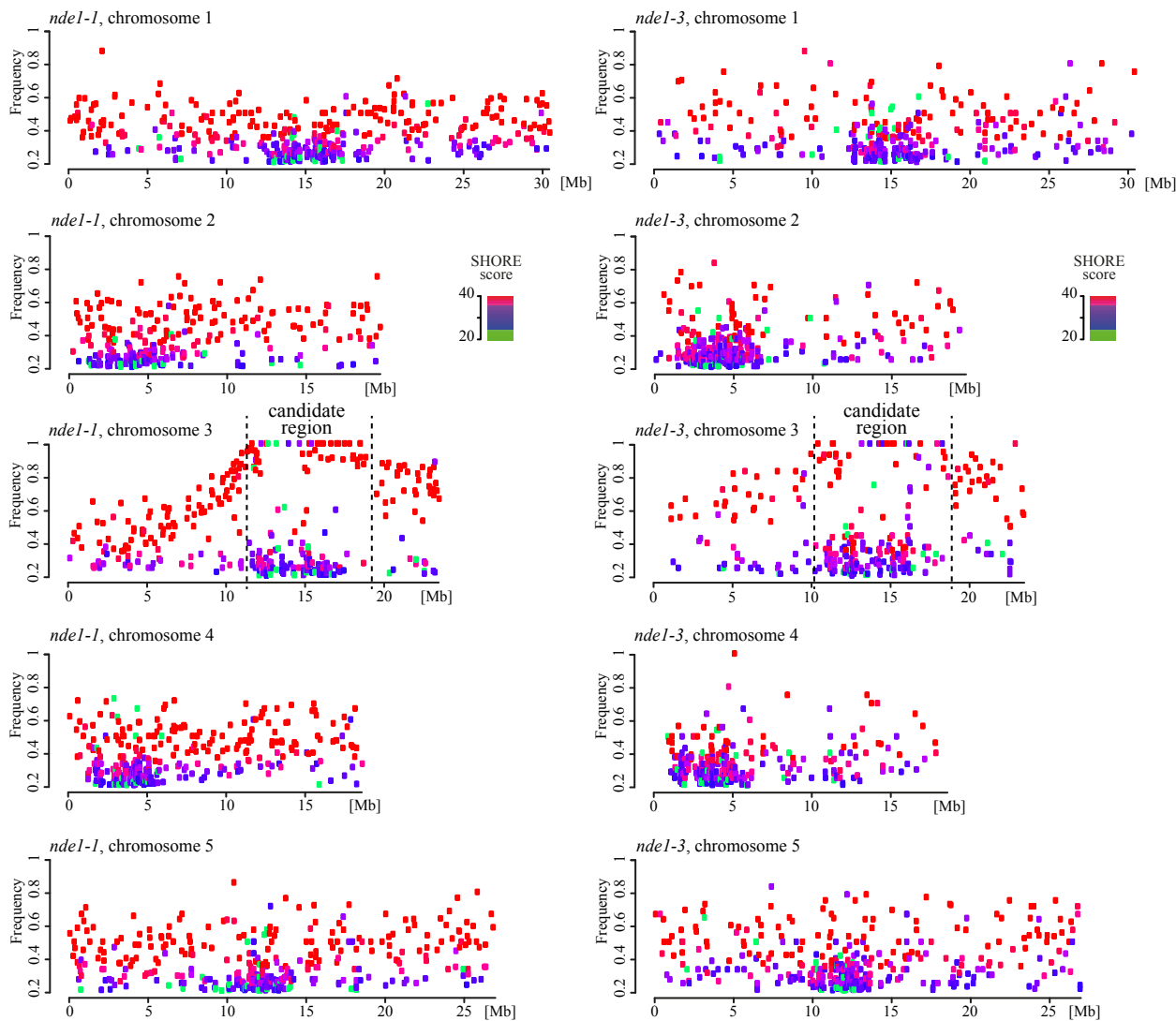

Supplement: S5 Fig — BC1-F2 bulked segregant DNAs from nde1-1 and nde1-3 were Illumina-sequenced and allele frequency estimates at EMS changes displayed using SHOREmap backcross after subtraction of SNPs from the parental EDS1-YFPNLS #A3 line. (PDF) [file pgen.1005990.s009.pdf]

Supplemental Figure S6

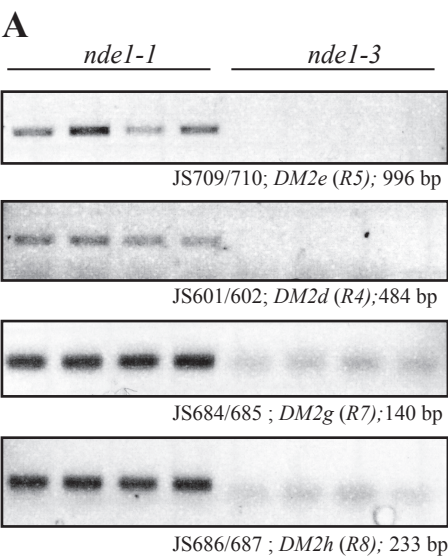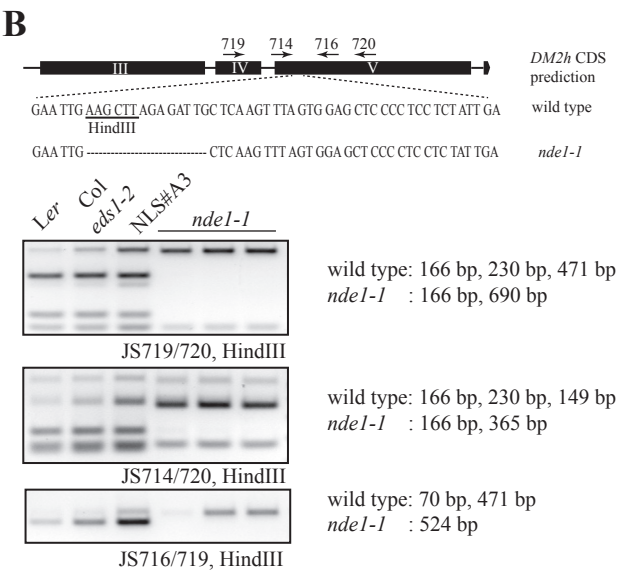

Supplement: S6 Fig — A. Amplicons within RPP1-likeLer genes DM2d, e, g and h (R4, R5, R7 and R8) were made using the indicated primer combinations (S4 Table) on four independent DNA preparations from nde1-1 or nde1-3. Optimized annealing temperatures and non-saturating cycling conditions were used to avoid non-specific amplification from ortholog-encoding DM2a-DM2h (R1-R8) loci. B. Detection of nde1-1 with CAPS markers. PCR amplicons were generated using the indicated primer combinations, digested with HindIII and resolved on agarose gels. Due to non-specific amplification from ortholog-encoding DM2a-DM2g (R1-R7) loci, some uncleaved PCR product is also visible in controls, but the wild type DM2h (R8)-specific cleavage product is never detected in nde1-1. Primer positions and the HindIII restriction polymorphism are indicated in a scheme. Expected HindIII fragments from wild-type and nde1-1 DNA are shown next to agarose gel images. (PDF) [file pgen.1005990.s010.pdf]

Supplemental Figure S7

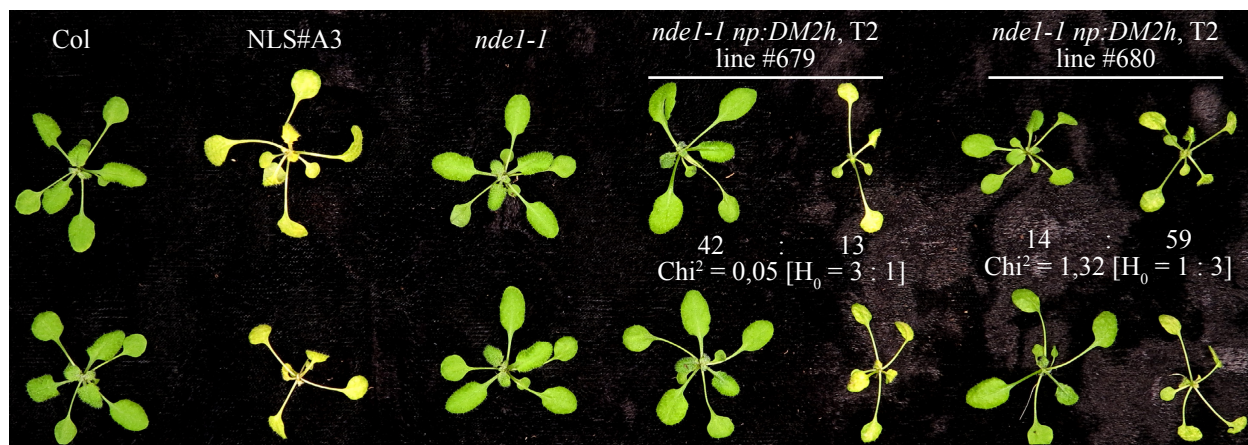

Supplement: S7 Fig — nde1-1 mutant plants were transformed with a genomic fragment containing DM2h under control of its native regulatory sequences. T2 plants and control lines were germinated at 22°C (7d), transferred to 28°C (12d) and shifted to 18°C for 10d. In both transgenic lines, necrotic seedlings were observed and counted. Segregation ratios indicate complementation of the nde1-1 mutant phenotype by single T-DNA insertions in both lines. (PDF) [file pgen.1005990.s011.pdf]

Supplemental Figure S8

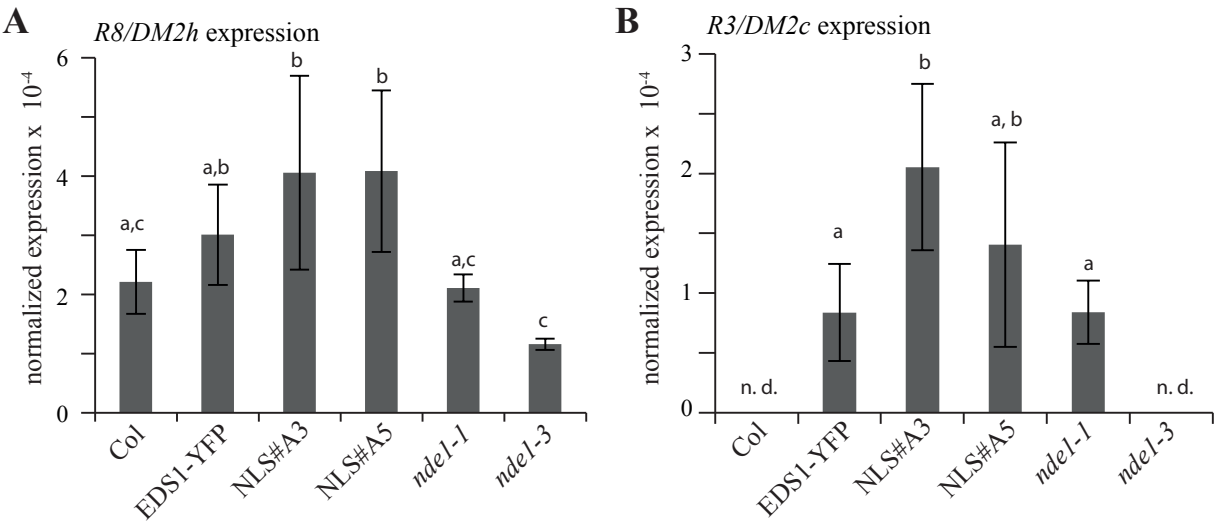

Supplement: S8 Fig — DM2h (A) and DM2c (B) expression was measured by qRT-PCR on the same samples used in Fig 6D. Standard deviation of ≥ 3 biological replicates is shown. Letters indicate statistically significant differences (ANOVA, Fisher’s LSD Post-hoc test, p < 0,05). (PDF) [file pgen.1005990.s012.pdf]

Supplemental Figure 9

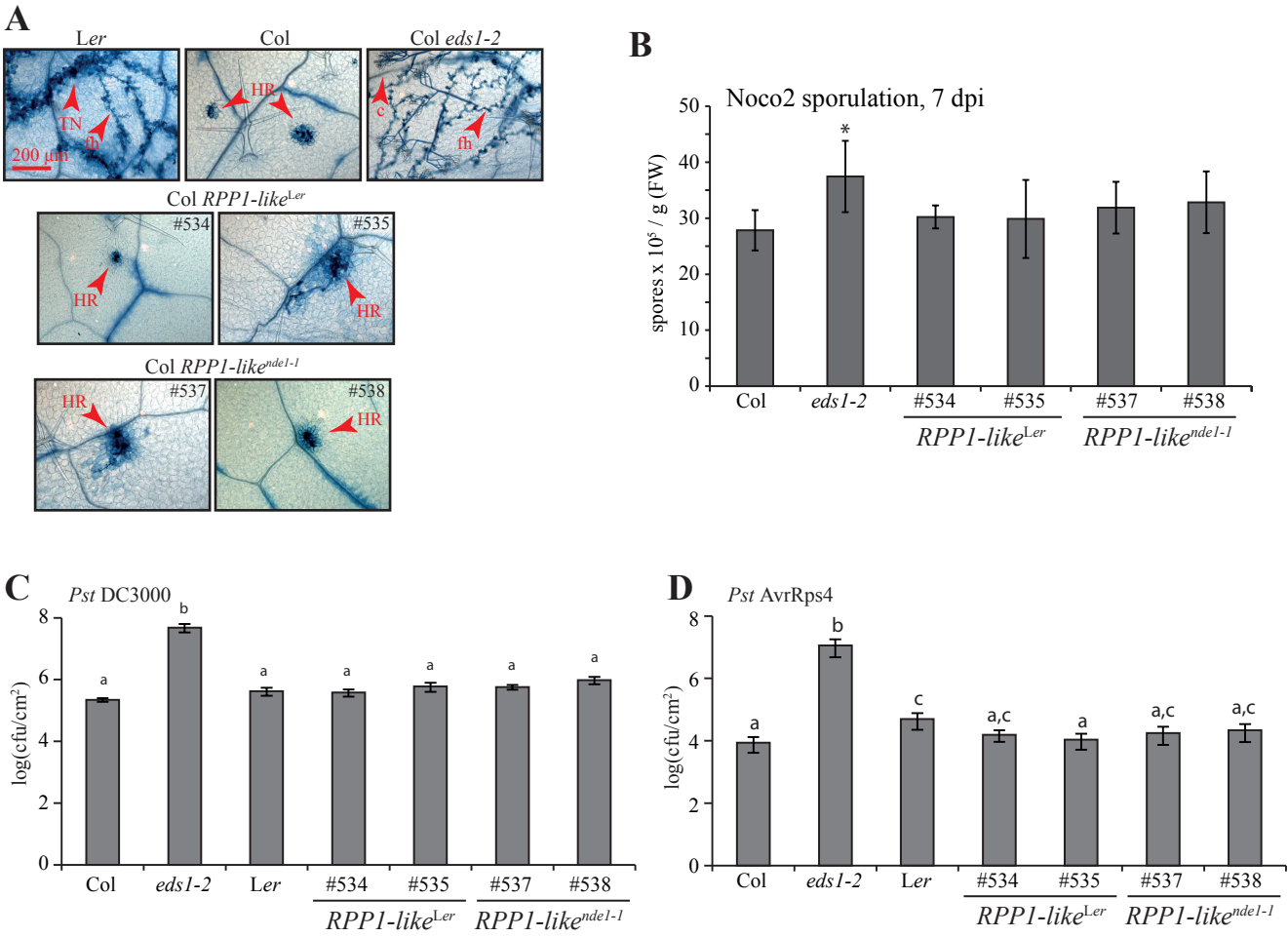

Supplement: S9 Fig — A. 2,5-week-old plants of the indicated genotypes were infected with Hpa Cala2 and first true leaves stained with Trypan Blue at 6 dpi. Representative micrographs of infection sites are shown. fh—free hyphae; TN—trailing necrosis; HR—hypersensitive response. B. As in A, but plants were infected with Hpa Noco2 and sporulation determined at 7 dpi. Error bars indicate standard deviation of four biological replicates. Asterisk indicates statistically significant difference to Col (Student’s t-test, p < 0,05). C. Bacterial growth of Pst DC3000 bacteria at 3 dpi on 5-week-old spray-infected plants of the indicated genotypes. Error bars indicate standard deviation of 8 biological replicates. Letters indicate statistically significant differences (ANOVA, Fisher’s LSD Post-hoc test, p < 0,01). D. Bacterial growth assays as done in C but plants were infected with Pst AvrRps4 bacteria. (PDF) [file pgen.1005990.s013.pdf]

# Supplemental Figure S10

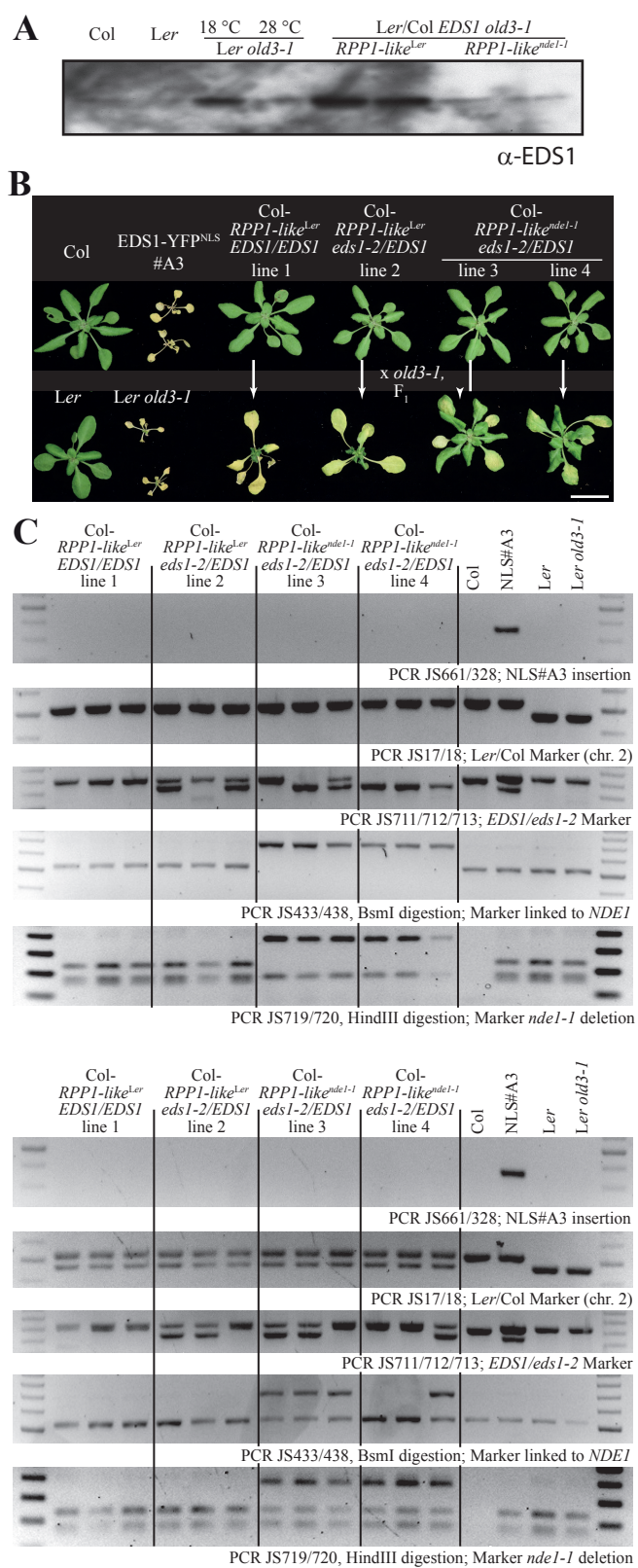

Supplement: S10 Fig — A. Immunoblot analysis of total protein extracts from the indicated genotypes separated by SDS-PAGE and probed with α-EDS1 antibodies. Protein samples were prepared from the same plants at the same stage as in Fig 8B. B. Col-RPP1-likeLer and Col-RPP1-likende1-1 NILs were crossed to Ler old3-1 mutant plants, F1 plants and controls grown at 23°C and phenotypes documented after 3 weeks. Col and Ler plants are healthy, but old3-1 and EDS1-YFPNLS #A3 plants are necrotic. F1 plants from crosses of old3-1 with Col-RPP1-likeLer NILs, which are heterozygous for old3-1/OLD3 and homozygous for RPP1Ler, are also necrotic, although not as severely as old3-1 control plants. Autonecrosis is further reduced in F1 plants from crosses of old3-1 with Col-RPP1nde1-1, heterozygous both for old3-1/OLD3 and RPP1Ler/RPP1nde1-1 and thus lacking one copy of functional RPP1-likeLer DM2h (R8) compared to F1 plants from crosses with Col-RPP1likeLer. C. Genotyping of plants from (A). Primer combinations and loci queried by different genetic markers are indicated. Primer sequences are given in S4 Table. (PDF) [file pgen.1005990.s014.pdf]
