# Supplementary material for: Arabidopsis thaliana DM2h (R8) within the Landsberg RPP1-like Resistance Locus Underlies Three Different Cases of EDS1-Conditioned Autoimmunity
Source: PLoS Genet. 2016 Apr 15;12(4):e1005990. doi: 10.1371/journal.pgen.1005990 (PMC4833295; doi:10.1371/journal.pgen.1005990)
Supplement: S11 Fig — Amino acid sequence corresponds to the CDS prediction shown in Fig 7. The predicted TIR domain (SMART search) is underlined. LRRs were annotated according to [18] and refined manually. Consensus positions are aligned and marked in red. Additional features (predicted NLS and N-myristoylation motif, mutations in nde1 alleles) are boxed and indicated. (PDF) [file pgen.1005990.s015.pdf]

Supplemental Figure S11

predicted N-myristoylation motif

predicted bipartite NLS

001 M**G**SAMSLSCS**KRK**TTSQDVDSES**RKRRK**ICSTNDAENCRFIQDESSWKHP 50
051 WSLCVNVAAAAFTKFRFQQDNKYTKSSALSLPSPPTSVSRIWKHHVFPSF 100
101 HGADVRKTILSHILESFRRKGIDPFIDNNIERSKSIGHELKEAIKGSKIA 150
151 IVLLSKNYASSSWCLDELAEIMKCRELLGQIVMTIFYEVDPTDIKKQTGE 200
201 FGKAFTKTCKGKTKEYVERWRKALEDVATIAGEHSRNWRNEADMIEKIAT 250
251 DVSNMLNSFTPSRDFDGLVGMRAHMDMLEQLLRLDLDEVRMIGIWGPPGI 300
301 GKTTIARFLFNQVSDRFQLSAIIVNIRGIYPRPCFDEYSAQLQLQNQMLS 350
351 QMINHKDIMISHLGVAQERLRDKKVFLVLDEVDQLGQLDALAKETRWFGP 400
401 GSRIIITTEDLGVLKAHGINHVYKVKYPSNDEAFQIFCMNAFGQKQPHEG 450
451 FDEIAREVMALAGELPLGLKVLGSALRGKSKPEWERTLPRLKTSLDGNIG 500
501 SIIQFSYDGLCDEDKYLFLYIACLFKDELSTKVEEVLANKFLDVKQGLHV 550
551 LAQKSLISIDENSFYGDTINMHTLLRQFGRETSRKQFVYHGFTKRQLLVG 600
601 ERDICEVLSDDTIDSRRFIGIHLDLYKSEEELNISEKVLERVHDFHFVRI 650
651 DASFQPERLQLALQDLICHSPKIRSLKWYSYQNICLPSTFNPEFLVELHM 700
701 SFSKLRKLWEGTKQLRNLKWMDLSNSEDLKELPNLSTATN 740

LRR1 741 **L**EE**L**K**L**RD**C**SS**L**VE**L**PSSIEKLTS 764
LRR2 765 **L**QR**L**Y**L**QR**C**SS**L**VE**L**PSFGNATK 787
LRR3 788 **L**EE**L**Y**L**EN**C**SS**L**EK**L**PPSINANN 810
LRR4 811 **L**QQ**L**S**L**IN**C**SR**V**VE**L**PAIENATN 833
LRR5 834 **L**QV**L**D**L**HN**C**SS**L**LE**L**PPSIASATN 857
LRR6 858 **L**KK**L**D**I**SG**C**SS**L**VK**L**PSSIGDMTN 881
LRR7 882 **L**DV**L**D**L**SN**C**SS**L**VE**L**PININLKS 904
LRR8 905 **F**LA**V**N**L**AG**C**SQ**L**KS**F**PEISTKIFTDCYQRMSR 936

C -> Y in *nde1-175*

LRR9 937 **L**RD**L**R**I**NN**C**NN**L**VS**L**PQLPDS 957
LRR10 958 **L**AY**L**Y**A**DN**C**KS**L**ER**L**DCCFNNPEIS 982

983 LNFPKCFKLNQEARDLIMHTTCINATLPGTQVPACFNHRATSGDSLKIKL 1032

R -> C in *nde1-150*

1033 KESSLPTTLRFKACIMLVKVNEEMSSDLKSMSFDPMRVDIVIRDEQNDLK 1082

W -> STOP in *nde1-13*

1083 VQCTPSYHFINHFIISTEHIYTFELEVEEVTSTELVFEFTLDKESNWKRN 1132
1133 WKIGECGILQRETRSLRRSSSPDLSPESSRVSSCDHC 1169
